# The environmental impact of telemonitoring vs. on-site cardiac follow-up: a mixed-method study

**DOI:** 10.1093/ehjdh/ztaf012

**Published:** 2025-02-26

**Authors:** Egid M van Bree, Lynn E Snijder, Sophie ter Haak, Douwe E Atsma, Evelyn A Brakema

**Affiliations:** Department of Public Health and Primary Care, Leiden University Medical Center, Albinusdreef 2, PO Box 9600, 2300 RC Leiden, the Netherlands; Department of Surgery, Maastricht University, Universiteitssingel 50, PO Box 616, 6200 MD Maastricht, the Netherlands; Center for Sustainable Healthcare, Amsterdam University Medical Center, Amsterdam, the Netherlands; Department of Public Health and Primary Care, Leiden University Medical Center, Albinusdreef 2, PO Box 9600, 2300 RC Leiden, the Netherlands; Department of Cardiology, Leiden University Medical Center, Leiden, the Netherlands; Faculty of Industrial Design Engineering, Delft University of Technology, Delft, the Netherlands; Department of Public Health and Primary Care, Leiden University Medical Center, Albinusdreef 2, PO Box 9600, 2300 RC Leiden, the Netherlands

**Keywords:** Carbon footprint, Sustainable healthcare, Life cycle assessment, Remote patient monitoring, Digital healthcare, eHealth

## Abstract

**Aims:**

Digital health technologies are considered promising innovations to reduce healthcare’s environmental footprint. However, this assumption remains largely unstudied. We compared the environmental impact of telemonitoring and care on site (CoS) in post-myocardial infarction (MI) follow-up and explored how it influenced patients’ and healthcare professionals’ (HPs) perceptions of using telemonitoring.

**Methods and results:**

We conducted a mixed-method study; a standardized life cycle assessment, and qualitative interviews and focus groups. We studied the environmental impact of resource use per patient for 1-year post-MI follow-up in a Dutch academic hospital, as CoS or partially via telemonitoring. We used the Environmental Footprint 3.1 method. Qualitative data were analysed using Thematic Analysis. The environmental impact of telemonitoring was larger than CoS for all impact categories, including global warming (+480%) and mineral/metal resource use (+4390%). Production of telemonitoring devices contributed most of the environmental burden (89%). Telemonitoring and CoS achieved parity in most impact categories at 65 km one-way patient car commute. Healthcare professionals and patients did not consider the environmental impact in their preference for telemonitoring, as the patient’s individual health was their primary concern—especially after a cardiac event. However, patients and HPs were generally positive towards sustainable healthcare and willing to use telemonitoring more sustainably.

**Conclusion:**

Telemonitoring had a substantially bigger environmental impact than CoS in the studied setting. Patient commute distance, reuse of devices, and tailored use of devices should be considered when implementing telemonitoring for clinical follow-up. Patients and HPs supported these solutions to enhance sustainability-informed cardiovascular care as the default option.

## Graphical Abstract

**Graphical Abstract:**
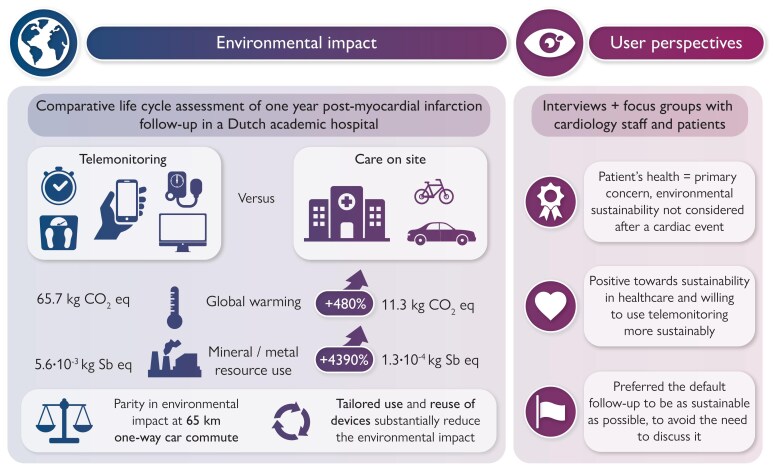

## Introduction

Human-induced environmental change presents a fundamental threat to global health this century.^1^ Heat stress, air pollution, and weather extremes negatively influence cardiovascular health, reinforcing global and regional health inequities.^2–4^ Adaptation and mitigation of these risks is urgently needed. The importance of reducing activities that contribute to environmental harm, such as the combustion of fossil fuels, was therefore underlined by major medical journals and the World Health Organisation.^5,6^

The healthcare sector contributes notably to environmental problems. Globally, it accounts for 4–5% of annual carbon emissions and in high-income countries for approximately 6–8%.^7^ In the Netherlands, the healthcare sector also contributes 13% of annual resource usage.^8^ Several European countries have therefore set targets to rapidly reduce the carbon emissions of the healthcare sector by 55% in 2030, in line with EU-wide Green Deal policies.^9,10^ Patient and staff commutes are a focus for impact mitigation, considering their significant contribution to carbon emissions.

Digital health technologies are considered promising innovations in cardiovascular care; not only to increase the efficiency and efficacy of disease management,^11,12^ but also to reduce travel-related emissions.^13,14^ For example, introduction of telemonitoring in post-myocardial infarction (MI) follow-up, which was reported to bear similar health outcomes, be cost-effective, and reduce the number of physical outpatient clinic visits.^15,16^ However, standardized assessments of digital health technologies’ environmental impact are rare and the impact of device production and digital infrastructure were frequently left out of scope.^17,18^

A life cycle assessment (LCA) is the routine method to investigate the environmental impact of products, processes, and services from ‘cradle to grave’.^19^ Only several LCAs of digital health technologies in clinical care exist,^20–23^ of which none are related to telemonitoring. Moreover, there is a knowledge gap how patients and healthcare professionals (HPs) perceive the importance of telemonitoring’s environmental impact when deciding if and how to use it. A combination of quantitative and qualitative research can be vital to translate evidence into systemic and organisational change.^24,25^ Therefore, to harness telemonitoring’s use for more sustainability-informed cardiovascular care, a better understanding of user perspectives is required.^14,26,27^

Given these gaps in the literature, the aim of this study was twofold: to compare the environmental impact of post-MI follow-up as care on site (CoS) alone and follow-up that included telemonitoring; and to explore how telemonitoring’s environmental impact influences patients’ and HPs’ perceptions of its use in follow-up post-MI and post-catheter ablation (CA).

## Methods

### Study design

We chose a mixed-method study design to deepen understanding of telemonitoring’s environmental impact and aid in its translation into sustainability-informed cardiovascular care.^24,25^ We conducted a comparative LCA of post-MI follow-up in line with international standards regarding the conduct and interpretation of LCAs (ISO14040/44) and a related transparency checklist for reporting (see Supplementary material online, *Supplement A*);^28,29^ combined with qualitative interviews and focus groups among cardiology patients and HPs using telemonitoring. Partially, data were based on anonymous clinical data from a preceding randomized clinical trial.^16^ Where applicable, we followed the COREQ guidelines for reporting of qualitative studies. Ethical approval was waived by the authorized hospital review committee (file number:24–033).

### Setting and comparability

Data were collected between March 2023 and January 2024 at the cardiology department of the Leiden University Medical Center, an academic hospital in the Netherlands. The majority of patients (>80%) are managed in dedicated care pathways, including post-MI and post-CA follow-up. Pathways are coordinated by nurse practitioners, specialized in specific cardiac conditions. Cardiologists are accountable for the organization of care, but are not usually involved in regular follow-up. Since 2013, telemonitoring using devices for non-invasive home measurements has gradually been introduced as a preferred way of working at the department. To facilitate the logistics of distribution and answer patients’ telemonitoring-related non-medical questions, a separate support office including patient-facing technical support staff was founded.

The preceding randomized clinical trial included 200 patients with ST-segment elevation MI or non-ST segment acute coronary syndrome, allocated to follow-up as CoS alone (*n* = 100) or including telemonitoring (*n* = 100, hereafter simply referred to as ‘telemonitoring’).^16^ There were no substantial differences in baseline characteristics between groups (median age 59.1 vs. 60.1, BMI 27.1 vs. 27.1, ST-elevation 78% vs. 79%, and hypertension 37% vs. 40%). After 1-year follow-up, there were no significant differences in blood pressure control, satisfaction with care, hospitalizations, or mortality. We therefore assumed clinical equipoise.

### Follow-up protocol

The protocolised number of diagnostic tests and devices used for post-MI follow-up had been slightly altered compared with the clinical trial. At the time, CoS consisted of four physical appointments (1, 3, 6, and 12 months) and diagnostic tests at the hospital: four electrocardiograms and blood pressure measurements, two blood tests, two cardiac ultrasounds, and one Holter monitoring. Telemonitoring consisted of two physical appointments (3 and 12 months), two virtual appointments (1 and 6 months), and diagnostic tests at the hospital: two electrocardiograms, one blood test, two cardiac ultrasounds, and one Holter monitoring.

Telemonitoring included electrocardiograms (and pedometer data) using a smartwatch (Scanwatch, Withings), blood pressure measurements using a wireless monitor (BPM Connect, Withings), and body weight measurements using a digital scale (Body, Withings). Measurements were ideally performed three times per week and monitored (bi)weekly by a local ‘service centre’ (trained medical students). When deviating from the patient’s regular trend, a notification email was sent to the nurse practitioner. They could: (i) consider the deviation irrelevant, (ii) wait until the next scheduled appointment to discuss it, or (iii) contact the patient. Data were not monitored continuously, nor a substitute for emergency care. Patients were instructed to contact the hospital in case of abnormalities or complaints. Further details regarding devices and follow-up are available in Supplementary material online, *Supplement B* and the preceding clinical trial.^16^

## Part 1—life cycle assessment

Life cycle assessment is a scientific, robust method, using an inventory of material and energy flows (e.g. raw material extraction and creation of printed circuit boards) required for a certain product or service (e.g. a blood pressure monitor) to quantify the resulting environmental impacts based on material- and resource-specific characterization factors. According to international standards,^28^ LCA consists of four phases: (i) a goal and scope definition; (ii) assembly of the inventory relevant to the subject of study; (iii) environmental impact assessment, based on a validated model of characterization factors; and (iv) interpretation of results, preferably including sensitivity and uncertainty analyses using probability distributions.^30^

### Goal and scope

**Figure 1:**
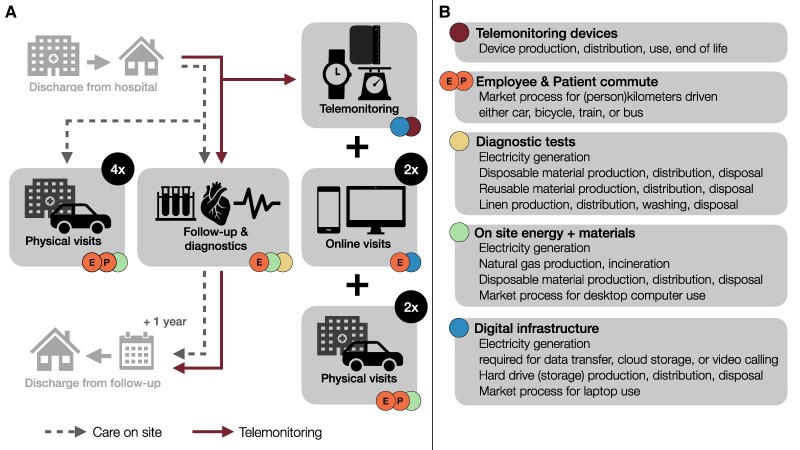
Overview of the post-myocardial infarction follow-up investigated in this study. (*A*) Elements of 1-year follow-up included in the analysis (grey rectangles) using either care on site or telemonitoring, including indication of visit frequency. (*B*) System boundaries of products, processes, and services included in the LCA; coloured dots indicate where in the follow-up categories occur. Employees still commuted to the hospital when performing online visits and diagnostic tests, which is therefore included in the analysis.

To compare CoS and telemonitoring, we studied all resources required per patient for planned care during 1-year post-MI follow-up (the ‘functional unit’). Resources considered in this LCA were: telemonitoring devices, patient and staff commute, diagnostic tests, outpatient clinic energy use, materials used for CoS, and the digital infrastructure required for telemonitoring (*Figure 1*). The production of the in-hospital devices used for diagnostic tests and hospital infrastructure were not included, since the impact was considered marginal after allocation to a single patient. Neither were individual patients’ pharmaceuticals or rehabilitation programmes considered. We included data for the entire life cycle, from raw material extraction to disposal or recycling (cradle-to-grave).

### Data collection

We examined all products, processes, and services relevant to the follow-up and categorized them into groups (*Table 1*). Required quantities of products and processes (foreground data) were based on cardiology protocol analysis, interviews with cardiology staff and hospital engineers, preceding LCA-studies, and IT-expert consultation. Environmental impact data regarding the smartwatch and body weight scale were obtained from environmental impact reports of the manufacturer. We verified data by fully disassembling both devices to determine material composition and weight, and independently calculated the corresponding environmental impact. For the blood pressure monitor, we primarily used our own disassembly data. Information regarding energy use and employee commute was based on the hospital’s reporting. The weight and composition of disposables and reusables for diagnostic tests were partially based on previous LCA work in another academic hospital. Details are listed in *Table 1* and Supplementary material online, *Supplement B*.

**Table 1:** Overview of data collection for the LCA

Group	Included in the analysis	Data collection
Telemonitoring devices	The production, distribution, use, and disposal of the smartwatch, body weight scale, and blood pressure monitor required for telemonitoring	Manufacturer environmental impact reporting for the year 2021, executed by external agency, mainly for smartwatch and body weight scale; complemented by own disassembly of all devices, examination of weights and composition, and preceding LCA-studies
Patient commute	The distance and means of commute of patients attending a physical consultation at the hospital	Preceding clinical trial and interviews with nurse practitioners
Employee commute	The distance and means of commute of employees involved in the care for a single patient; allocated based on the share of workload spent on a single patient	2021 hospital-wide audit of employee commute
Building energy use	The electricity consumption and natural gas usage for the outpatient clinic consultation room, including lighting, heating, and air ventilation; allocated based on the time required for a single patient	2022 annual hospital energy reporting, specifically reported energy use per m^2^ for the outpatient clinic (electricity) and entire hospital (natural gas)
Diagnostic tests	The material and energy requirements for electrocardiograms, trans-thoracic echocardiograms, Holter monitoring, phlebotomy, and blood tests	Quantity of tests based on cardiology protocols and interviews with staff; disposable and reusable product usage and device energy consumption based on previous LCA research in another Dutch academic hospital and preceding LCA-studies
Digital infrastructure	The data storage required for telemonitoring of an individual patient and the energy required for data transfer, cloud storage, site mirroring (= online availability on servers in close proximity), and video calling	Interviews with hospital IT-specialists for required data size, number of data transfers, and data storage location; complemented by preceding scientific studies regarding data transfer and consultation of external data engineering experts
On-site materials	The disposable gloves, examination table paper, and hand disinfection used during physical visits	Quantity based on interviews with nurse practitioners; material composition based on previous LCA in other Dutch hospital and preceding LCA-study

Details on means of data collection and LCA-modelling are provided in Supplementary material online, *Supplement A*.

**Table 2:** Environmental impact of post-MI follow-up using telemonitoring or care on site

	Global warming kg CO_2_ eq	Particulate matter formation Disease incidence	Water use m^3^ water	Mineral/metal resource use kg Sb eq	Fossil resource use MJ
**Telemonitoring**					
Smartwatch	31.9	8.6 E-7	4.5	1.3 E-3	511.0
Body weight scale	15.0	7.6 E-7	7.7	2.4 E-3	214.1
Blood pressure monitor	11.2	3.9 E-7	1.4	2.1 E-3	93.6
Patient commute	3.9	2.1 E-7	0.9	4.3 E-5	52.8
Employee commute	1.2	6.5 E-8	0.2	1.1 E-5	15.8
Building energy use	0.6	3.8 E-9	<0.1	6.2 E-7	10.2
Diagnostic tests	1.3	5.6 E-8	1.2	1.8 E-5	19.3
Digital infrastructure	0.5	8.5 E-9	0.3	4.8 E-6	12.4
On-site materials	0.1	5.1 E-9	<0.1	7.0 E-6	1.4
**Total**	**65**.**7**	**2.4 E-6**	**16**.**3**	**5.6 E-3**	**930**.**6**
**Care on site**					
Patient commute	7.7	4.2 E-7	1.8	8.7 E-5	105.6
Employee commute	1.0	5.8 E-8	0.2	1.0 E-5	14.1
Building energy use	0.6	3.8 E-9	< 0.1	6.2 E-7	10.2
Diagnostic tests	1.8	8.1 E-8	1.5	2.4 E-5	26.0
On-site materials	0.1	8.5 E-9	0.1	7.8 E-6	2.2
**Total**	**11**.**3**	**5.7 E-7**	**3**.**6**	**1.3 E-4**	**158**.**5**
**Difference**	**54**.**4**	**1.8 E-6**	**12**.**8**	**5.7 E-3**	**777**.**9**
(Telemonitoring vs. CoS)	(+480%)	(+310%)	(+360%)	(+4390%)	(+510%)

Environmental impact is presented as the average value for each of the five available impact indicators of the (adjusted) Environmental Footprint 3.1 method. Values marked in bold indicate the total environmental impact per care type (i.e. telemonitoring or care on site) and the difference between the two.

### Data analysis

We modelled the data in SimaPro LCA-software v9.5.0.1 (PRé Sustainability, the Netherlands). Foreground data were combined with generic ‘background data’: information on other life cycle stages such as the production, shaping, and incineration of plastics. These were derived from the ecoinvent v3.9 database (Ecoinvent, Switzerland),^31^ using cut-off by classification market processes (i.e. including transport processes). For electricity generation and vehicles, more recent national datasets were used (including e.g. production and maintenance of vehicles).^32,33^ Details are listed in Supplementary material online, *Supplement B*.

We performed the impact assessment using the adjusted Environmental Footprint method v3.1 in SimaPro, which aligned with the provided device manufacturer information and European regulations.^34^ Accordingly, we reported the following environmental impact indicators: global warming in kg carbon dioxide equivalents (kg CO_2_ eq), particulate matter formation in cumulative change in disease incidence per kg of PM_2.5_ or precursors (disease incidence), fossil resource use in megajoules, mineral/metal resource use in kg antimony equivalent (kg Sb eq), and water use in m^3^. Differences between telemonitoring and CoS were calculated as percentage change of the total environmental impact. For each analysis group, contributions to the total environmental impact were determined per impact indicator.

To verify the robustness of findings and explore impact mitigation strategies, we performed sensitivity analyses to test the effect of reuse and tailored use of devices, such as leaving out the body weight scale or using a patient’s own, equivalent smartwatch. A maximum reuse for three consecutive patients was assumed, striking a balance between manufacturer’s environmental impact data and consulted nurse practitioners’ and support staff’s experience with the condition of devices after preceding patient use. In addition, we tested the effects of assumptions and database choices in the LCA model. As uncertainty analysis, we performed Monte Carlo simulations using 1000 runs for modelled ranges of foreground data and pedigree matrix-computed ranges of background data.^35^ We used our own device disassembly data for the uncertainty analysis, since ranges for manufacturer data were unavailable.

## Part 2—qualitative research

### Recruitment

We primarily recruited participants for interviews and focus groups by distributing a short online survey. A purposive sample of cardiologists, nurse practitioners, and patient-facing technical support staff directly involved in care pathways including telemonitoring received invitational emails from the main investigator (E.M.v.B.). Patients in post-MI or post-CA follow-up were recruited via a one-page invitation letter containing a link and QR-code to the survey, distributed by patient-facing technical support staff upon device distribution and by nurse practitioners during outpatient visits. Patients willing to participate but unable to access the survey were contacted via phone. The survey was anonymous, unless participants shared their email address to continue participation—which was rewarded with a 25-euro gift card. The survey invitation and introduction information did not clarify environmental sustainability as the subject of study to limit potential self-selection bias.

Due to experienced difficulty recruiting post-MI patients after 1 month of survey availability, nurse practitioners alternatively asked patients directly during outpatient visit whether the investigator could contact them via phone. Patients willing to participate were directly scheduled for an interview or focus group and did not take part in the survey. We planned not to conduct more than 15 interviews and 4 focus groups—which we expected to suffice for data saturation.

### Data collection

We performed interviews and focus groups between September 2023 and January 2024. Conversations were semi-structured, using a predefined topic list including Theoretical Domains Framework (TDF) constructs to probe for barriers and facilitators to explicitly discuss environmental impact when considering telemonitoring (see Supplementary material online, *Supplement C*).^36^ Participants were asked to base their answers on their own experience and were not informed of the study’s main subject of interest. We first openly explored perspectives regarding the use of telemonitoring and thereafter questioned how the environmental impact of telemonitoring had—or could have—influenced its use for follow-up. E.M.v.B. conducted all interviews (30–45 min) and focus groups (45–60 min), supervised by an experienced qualitative researcher (E.A.B.). Interviews were on-site or online, depending on the participant’s preference. Focus groups were on-site and joined by a second observant researcher. Conversations were audio recorded and transcribed verbatim in Microsoft Teams (Microsoft, USA). E.M.v.B. reviewed transcript accuracy and manually adjusted where necessary before analysis. Intermediate findings were used as input for follow-up questions in later interviews.

### Data analysis

We coded transcripts primarily using inductive coding to allow for an open exploration of relevant findings, supported by a deductive TDF-derived structure. Two junior researchers independently coded all transcripts and discussed after every transcript until consensus was achieved, supervised by E.M.v.B. as deciding third if necessary. Following the Framework Approach, codes and quotations were charted into data matrices to support the interpretation of findings.^37^ E.M.v.B., the junior researchers, and E.A.B. identified main themes using Thematic Analysis through intermediate and final analysis meetings.^38^ Two external researchers with extensive experience were consulted to validate the interpretation and structuring of findings.

### Reflexivity statement

The team consisted of researchers with diverse backgrounds (medicine, psychology, and healthcare management) and varying levels of experience. None of the interviewers were part of the cardiology department and this was communicated to participants. E.M.v.B. had previously spoken to several HPs for LCA data collection, yet had not discussed the content of the interview. Both E.M.v.B. and E.A.B. are involved in a national environmentally sustainable healthcare network. Whereas this background was helpful in designing and contextualizing the study, it may have influenced the interviews or focus groups by unconsciously responding more positively to comments in favour of sustainability. Involvement of the junior researchers, who had no previous affinity with the subject, helped to reduce such bias in coding and analysis.

## Results

### Part 1—life cycle assessment

As per protocol, on average two physical consultations were replaced by virtual visits, in addition to two fewer outpatient electrocardiogram recordings and one fewer phlebotomy and diagnostic blood tests. Average commute distance of patients was 7 km one way, which 50% of patients travelled by car and others by public transport or by bicycle. Every patient was allowed to keep the devices after completion of the 1-year follow-up.

### Environmental impact

**Figure 2:**
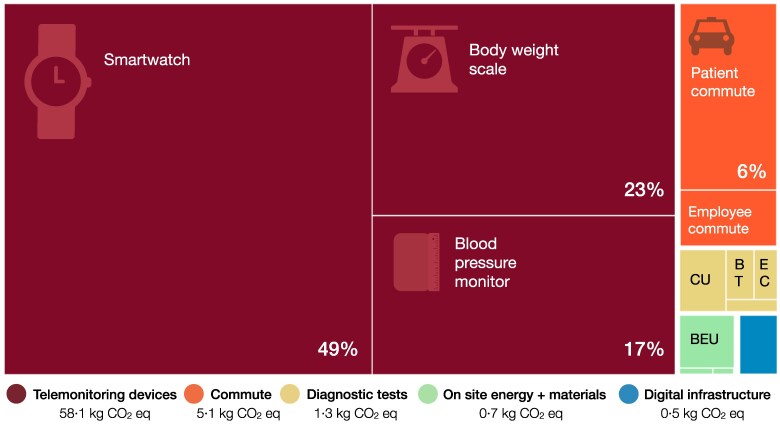
Treemap of global warming caused by telemonitoring for post-MI follow-up. Environmental impact is presented as a relative contribution (%) of individual resource groups to the total global warming caused by post-MI follow-up including telemonitoring. A comparative overview of care on site is not included, but can be found in Supplementary material online, *Supplement D*. Coloured categories indicate the resource groups included in the assessment (as defined in *Table 1*). Values ≤2% not indicated. CU, cardiac ultrasound; BT, blood tests; EC, electrocardiogram; BEU, building energy use.

The environmental impact of post-MI telemonitoring follow-up was higher than CoS in all five impact categories (*Table 2*). Global warming caused by telemonitoring was 65.7 kg CO_2_ eq compared with 11.3 kg CO_2_ eq for CoS (+480%). This roughly equals emissions of 437 and 75 km of driving an average Dutch petrol-fuelled car.^33^ The largest difference was observed for mineral/metal resource use (+4390%) and the smallest difference for particulate matter formation (+310%). For telemonitoring, the majority of the environmental impact was caused by device production (89% of global warming, *Figure 2*). Depending on the impact category, either the smartwatch or the scale caused most environmental impact. For CoS, patient car commute contributed most environmental impact (68% of global warming). Detailed results for additional impact categories are reported in Supplementary material online, *Supplement D*.

### Use strategies

**Figure 3:**
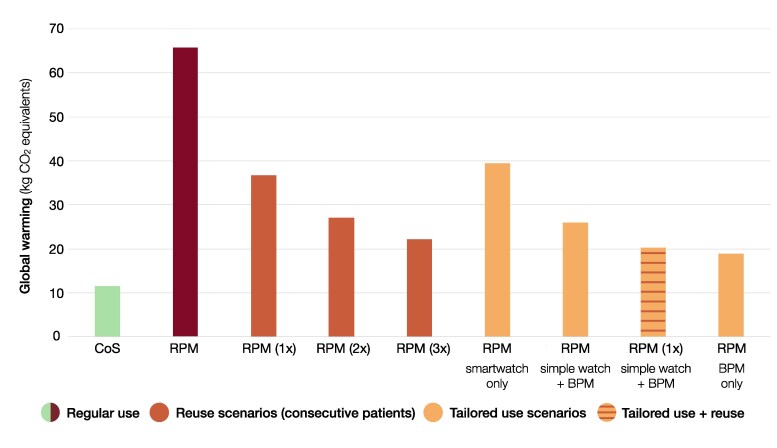
Effect of reuse and tailored use on the global warming caused by post-MI follow-up. Environmental impact is presented as global warming (kg CO_2_ eq) caused by post-myocardial infarction follow-up using care on site or telemonitoring. The effects of different scenarios are compared. CoS, care on site; RPM, telemonitoring; BPM, blood pressure monitor.

Tailored use or reuse scenarios of devices reduced the difference in incurred global warming between telemonitoring and CoS (*Figure 3*). Similarly, the smartwatch could be exchanged for a ‘simple watch’ (7.0 kg CO_2_ eq), which still includes an electrocardiogram recorder and pedometer. Other environmental impact categories showed similar results and differences remained largest for mineral/metal resource use (see Supplementary material online, *Supplement D*). Telemonitoring scenarios of device reuse up to three times and use of only a new blood pressure monitor still caused more global warming than CoS (21.4 and 18.1 kg CO_2_ eq respectively).

### Commute distance

**Figure 4:**
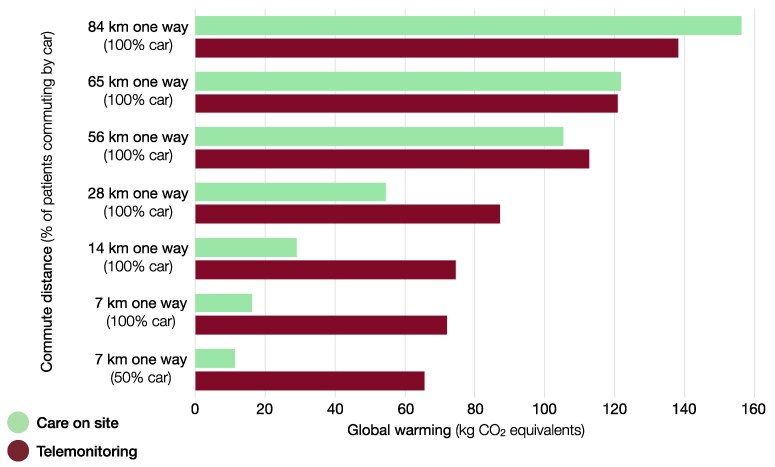
Effect of patient commute on the global warming caused by post-MI follow-up. Environmental impact is presented as global warming (kg CO_2_ eq) of the care pathway for post-myocardial infarction follow-up using telemonitoring or care on site. The effects of different commute distances and modes of travel (% car commute) are compared. The bottom scenario corresponds with the study setting (7 km one-way, 50% car commute).

Patient commute distance and mode of transport significantly influenced the environmental impact of follow-up, compared with the study scenario. Telemonitoring and CoS achieved parity for their contribution to global warming at one-way patient car commute distances of approximately 65 km (*Figure 4*). For longer commute distances, telemonitoring contributed less than CoS—not considering redistribution or tailored use strategies. Other environmental impact categories showed similar results, except for mineral/metal resource use, which remained higher (+240%) even at 84 km one-way car commute (see Supplementary material online, *Supplement D*).

### Database choices

Sensitivity analysis of database choice for types of vehicles in use mainly affected the environmental impact of the CoS scenario and reduced the commute distance of being at parity with telemonitoring to approximately 50 km one-way car commute (see Supplementary material online, *Supplement D*). Database choices for electricity generation and power efficiency of digital data transfer had minor effects on the comparison. Uncertainty analysis using own disassembly data confirmed that telemonitoring contributed significantly more to global warming, particulate matter formation, fossil resource use, and mineral and metal resource use than CoS in the study setting (see Supplementary material online, *Supplement D*).

## Part 2—qualitative research

In total, 8 HPs and 12 patients participated in interviews or focus groups: cardiologists (*n* = 2), nurse practitioners,^4^ patient-facing technical support staff,^2^ and patients in post-MI or post-CA follow-up (4 and 8 respectively; of whom 6 completed the survey). In addition, 2 cardiologists and 10 patients completed the online survey and 5 patients were contacted via phone, but were not willing to participate in the research. All patients had successfully used or were successfully using telemonitoring.

### Both healthcare professionals and patients preferred telemonitoring

The majority of HPs and patients considered telemonitoring to offer high quality care. Healthcare professionals valued the availability of additional blood pressure measurements and electrocardiograms with telemonitoring. Moreover, they could discuss the patient’s lifestyle and physical activity based on pedometer data and body weight (*Box 1*). Patients experienced reassurance regarding their health due to additional measurements, and felt more in control of their own health. Some perceived increased self-efficacy for lifestyle change. Both HPs and patients mentioned that telemonitoring might be unsuitable for people with lower digital literacy, yet expected this number to be limited.

Box 1 **Themes with their illustrating key quotes**

**Both Healthcare professionals and patients preferred telemonitoring**

Healthcare professional *5, nurse practitioner: ‘It’s in the patient’s interest that we can monitor them better. To monitor the trend, rather than snapshots that are frequently distorted by stress or white coat hypertension. And that we can objectify complaints more easily which only occur sporadically, based on the ECG or hypotension.’*

Healthcare professional *3, nurse practitioner: ‘Research and guidelines increasingly emphasize the importance of lifestyle for patients with cardiac arrhythmia. So, if someone is overweight and has a BMI larger than 25, they get a scale. The watch has a pedometer, so we give patients the instruction to be more active. This allows me to discuss it during follow-up visits.’*

*Patient 2, MI: ‘Especially after a heart attack, you’re quite insecure. In the sense that you think: maybe I’ll experience it again […] So, at that moment, telemonitoring is a really good solution. Some reassurance, I have to say.’*

*Patient 11, CA: ‘It gives a very pleasant feeling to know that your heart rate is normal and that it is being registered. In my opinion, that’s the big advantage of the smartwatch. I don’t have to call the hospital every week to ask: am I doing alright? I know that, apparently, I’m doing alright.’*

**The environmental impact was not considered in their preference**

Healthcare professional *2, cardiologist: ‘It’s always a priority that care is safe and effective, because that’s what I’m held accountable for. If I don’t guarantee that, I might even be punished. That doesn’t go for environmental sustainability. So that makes it less important.’*

Healthcare professional *5, nurse practitioner: ‘In the end, everything we do is centred around the patient and the patient’s interest. Of course, environmental sustainability is also something that’s in the interest of the patient. Yet, I consider it most important that it’s clear for the patient that their questions, complaints, test results, and uncertainties are at the top of the agenda.’*

*Patient 9, MI: ‘When dealing with serious medical concerns, I consider the environment to be of secondary importance. […] At that moment, the first and only thing that I think of is: does it contribute to my medical condition and the care that I receive? Anything else comes after that and is less relevant for me at that moment.’*

*Patient 12, MI: ‘In my opinion, it’s quite alright not to mention it explicitly [environmental sustainability]. Whereas in the larger scheme of things it’s quite important to consider the burden to nature and the environment, it’s mostly irrelevant for patients who have just experienced a heart attack. […] I might even wonder: are you trying to hide something instead of convincing me with medical arguments that I consider most relevant at that moment. Whilst, when I think of an environmental argument now, I find it quite interesting.’*

**Suggestions to reduce the environmental impact of telemonitoring**

Healthcare professional *6, nurse practitioner: ‘If I see how easily we use and give everyone a box [telemonitoring], I doubt how sustainable that is. [laughs] Patients get a box with several devices and basically they get to keep them if they use them. Only seldomly one returns […].’*

Healthcare professional *4, nurse practitioner: ‘If someone already has a smartwatch and comes in with all their gear, then I won’t offer them the entire box [telemonitoring]. I will provide a blood pressure monitor, but not a pedometer with a watch—I simply take it out. So I adjust it according to the patient.’*

*Patient 6, CA: ‘Half a year from now I have another checkup […] and if all is well, my care will be transferred back to the general practitioner who won’t do anything with my ECGs and other data anyways … then someone else can have my smartwatch.’*

*Patient 3, CA: ‘For my watch—the battery is dead—they said: you can keep it. While I would actually say: bring it back. The same goes for the blood pressure monitor. I already had one at home, so now I have two. I think that’s quite a shame, right, for the environment.’*

**Discussing environmental sustainability in consultations**

Healthcare professional *8, support staff: ‘You only discuss the most practical things at the patient’s bedside or on the phone. Also because their attention span is very short—for everyone, actually. […] If I were to have a conversation to talk about the environmental impact of telemonitoring directly afterwards, I think I would lose them.’*

Healthcare professional *1, cardiologist: ‘[…] that I do wonder if it [telemonitoring] is actually more sustainable. So, why would I then convince people of environmental sustainability? And actually, I feel like that’s not what people are looking for. They come in to discuss the procedure of a catheter ablation, not to meet with a sustainability coach.’*

*Patient 7, CA: ‘I wouldn’t mind if they bring up the environment. It’s important. But at that moment, after all, you’re a physician and a patient. I mean, […] while I do consider the environment important, I wouldn’t at that moment. They can mention it, but I would probably not consider or react to it.’*

*Patient 4, CA: ‘[…] At the moment there’s a lot of attention for the environment. You read about it more frequently. It’s pointed out more frequently. So yes, those are things that make you more environmentally conscious.’*

HP, health professional; MI, post-myocardial infarction follow-up; CA, post-catheter ablation follow-up.

### The environmental impact was not considered in their preference

Most HPs and patients did not consider the environmental impact in their preference for telemonitoring. Patients’ health was their primary concern, especially after experiencing a cardiac event (*Box 1*). None of the patients had discussed or considered telemonitoring’s environmental impact, even if they generally valued environmental sustainability in their daily lives and/or society. Several nurse practitioners had occasionally considered telemonitoring’s environmental impact when choosing which devices to use for certain patients, or when defining department standards. In varying degrees, patients and HPs perceived a need to improve environmental sustainability in healthcare. Opinions differed regarding who should consider it and when. Most participants suggested to ensure and incorporate it by default—without explicitly involving patients.

### Suggestions to reduce the environmental impact of telemonitoring

Participants considered it relevant to reduce the environmental impact of clinical care. Healthcare professionals had contrasting ideas regarding how to reduce the environmental impact of telemonitoring. Some argued that telemonitoring’s device use was excessive and per definition unsustainable. Others assumed patients’ long-term health benefits to reduce care consumption, resulting in better patient and environmental outcomes. Both HPs and patients suggested reuse and more tailored use of devices based on individual patient characteristics to be acceptable and feasible (*Box 1*). Although some patients appreciated continued self-monitoring after follow-up, most indicated willingness to return devices if they could be used for another patient.

### Discussing environmental sustainability in consultations

**Figure 5:**
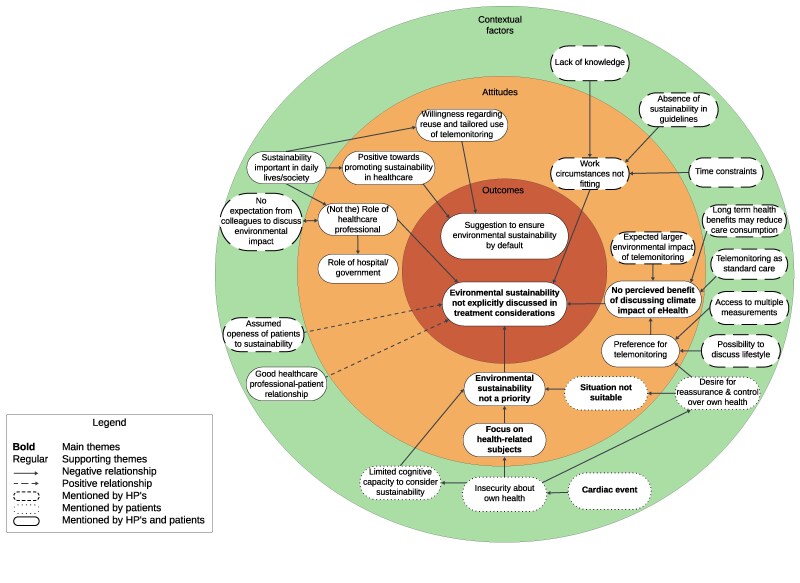
Connection of outcomes, attitudes, and contextual factors considering the environmental impact of telemonitoring. Identified themes are presented in corresponding circles: outcomes (inner circle), attitudes (middle circle), and contextual factors (outer circle).

Several barriers and facilitators influenced whether environmental sustainability was explicitly mentioned when discussing telemonitoring (*Box 1*, *Figure 5*). First, HPs and patients strongly prioritized topics related directly to the individual patient’s health. With limited time available for a consultation (indicated by HPs) or limited cognitive capacity (mainly indicated by patients) environmental sustainability was considered inexpedient to discuss. Consultations usually took place soon after a cardiac event, so patients wanted to focus on their health and expected HPs to do the same. Second, HPs and patients did not perceive a benefit to discuss environmental sustainability, as it would not alter their decision regarding its usage. Moreover, HPs who expected telemonitoring’s environmental impact to be larger, considered it an afterthought since it was the agreed standard care in the department. Healthcare professionals mentioned several additional barriers, yet were inconsistent in the perceived importance of those barriers: a lack of knowledge, doubts whether discussing sustainability was part of their professional role, and the absence of sustainability in guidelines. Facilitators were: perceived general importance of sustainability, assumed openness of the patient to the topic, and a more developed HP-patient relationship.

## Discussion

We conducted a mixed-method study, performing the first LCA of cardiac follow-up using telemonitoring devices; and the first qualitative study offering in-depth, complementary insights into how its environmental impact influenced patients’ and HPs’ perceptions regarding its use. We uncovered that post-MI telemonitoring follow-up had a substantially larger environmental impact than CoS in the studied setting, including global warming (+480%) and mineral/metal resource use (+4390%). Telemonitoring device reuse and tailored use substantially reduced its environmental impact. Depending on patients’ commute distance to the hospital, a similar or lower environmental impact than CoS could be achieved—although mineral/metal resource use remained higher, even at large commute distances. Healthcare professionals and patients did not consider the environmental impact in their preference for telemonitoring, as the patient’s individual health was their primary concern—especially after a cardiac event. However, patients and HPs were generally positive towards sustainable healthcare and were willing to use telemonitoring more sustainably.

Three recent LCAs unanimously documented a lower contribution to global warming of digital health technologies for outpatient consultations compared with CoS.^20,22,23^ In all cases, the difference originated from avoided patient commute (25–402 km of driving per patient) and low emissions (<1 kg CO_2_ eq) per virtual consultation. However, none of these studies considered the environmental impact of telemonitoring devices. The global warming caused by the use of digital health technologies in our study (54.4 kg CO_2_ eq) was therefore substantially higher. Other environmental impact categories, such as mineral/metal resource use, have not been reported before. Notably, average patient commute distances in our setting were relatively short (7 km), considering that it concerns a small and densely populated country. For longer one-way travel distances (>65 km), telemonitoring would have caused less global warming than CoS. While our findings therefore support an environmental benefit of telemonitoring to reduce patient commute in more remote settings, they also underline the need to evaluate the environmental impact of telemonitoring devices.

In the studied setting, the incurred environmental harm of telemonitoring for clinical follow-up could be substantially reduced (up to 78%) if devices were reused for consecutive patients or only distributed to patients without an own, equivalent device. In essence, these strategies align with key circularity principles that also apply to telemonitoring follow-up for other medical conditions: ‘refuse’—avoiding resource use for abundant devices; and ‘reuse’—minimising additional resource use per patient.^39^ Whilst the use of patients’ own wearables has not been validated in our setting, successful integration of patients’ smartwatch data for clinical telemonitoring was recently documented elsewhere.^40^ Moreover, reuse of devices was described previously in relation to cardiac implantable electronic devices,^41^ suggesting that suitability for telemonitoring using non-invasive measurements may be assumed if devices can be decontaminated and cleared of stored data.^39^ Selecting devices based on these criteria and collaborating with manufacturers to facilitate repair or remanufacturing of devices may yield further benefits.

To date, few studies have documented perspectives regarding the environmental impact of medical decisions, especially among patients.^42^ Three separate surveys in the Netherlands indicated that patients may be willing to choose a more environmentally friendly treatment when its environmental impact is addressed explicitly.^43–45^ Some patients even accepted slightly reduced treatment comfort or efficacy. However, we found that HPs and patients strongly prioritized health-related subjects, which may be explained by the severity of a cardiac event compared with the medical conditions in the surveys (shoulder pain and migraine). Furthermore, participants considered it inexpedient or unfeasible to explicitly discuss telemonitoring’s environmental impact. To avoid the need to discuss it, the majority preferred the default option (in this case: telemonitoring) to be as sustainable as possible and suggested reuse and tailored use of devices to be acceptable and feasible.

Strikingly, contrasting opinions emerged regarding the environmental impact of telemonitoring in the studied setting. Some HPs considered telemonitoring device use unsustainable per definition, whilst others hypothesized long-term health benefits—possibly reducing the impact of future care consumption. Notably, evidence regarding long-term benefits of telemonitoring in clinical follow-up is frequently technology- and disease-specific (e.g. heart failure) and can be equivocal.^12,46,47^ Therefore, one may question: when does a (health) benefit weigh up to a larger environmental burden? Where sufficient evidence is available, an integral multi-criteria decision analysis including environmental sustainability could support decision-making.^48^ In other cases, a professional debate could yield consensus to what extent different quality criteria justify implementation (e.g. staffing requirements, access to care, and environmental impact). As previously addressed in the literature,^49,50^ the environmental burden of healthcare can be (and in our opinion: should be) perceived as a scarce resource.

Strengths of this study were its mixed-method design, which deepened understanding of telemonitoring’s environmental impact and aids in its translation into sustainability-informed cardiovascular care;^24,25^ and the meticulous quantification of multiple environmental impacts using standardized LCA methodology. Notwithstanding these strengths, several limitations merit consideration when interpreting our findings. First, LCA impact allocation choices were made, especially regarding device use. Partial attribution to personal or continued use after follow-up would have resulted in a lower environmental impact. Given that patients received these devices for telemonitoring, we considered our choice (100% allocation) justified and analysed the effect of a reduced allocation in the sensitivity analysis—which did not alter our key findings. Second, we did not collect clinical data for other cardiac telemonitoring applications or suggested device use strategies. Whereas this would have broadened the applicability of findings, it would also have impaired feasibility to include detailed information regarding devices and digital infrastructure in the analysis—seldomly included in preceding studies.^17,18^ Moreover, we do believe underlying conclusions to be relevant to other telemonitoring follow-up using similar devices or taking place in similar settings. Third, results of the interviews may predominantly include sustainability-minded perspectives or be subject to social desirability bias when participants stated the importance of environmental sustainability. We limited this bias by only informing participants of the study’s purpose during participation. Fourth, all participating patients successfully used telemonitoring, which may have caused a biased perception of telemonitoring follow-up. Whereas patients with lower digital health literacy may have additional perspectives regarding the environmental sustainability of telemonitoring, this was not a focus of the current study and in HP’s experience this group was rather small.

We argue that future telemonitoring studies should (strongly) consider the environmental impact of their intervention in its clinical implementation. Our findings underline the importance of standardized LCA and thorough evaluation of the complete environmental impact, including telemonitoring devices, rather than back-of-the-envelope calculations of avoided car commute. Whereas it may not always be feasible to perform an LCA, we do believe that investigators can generally consider the impact of required resource groups (e.g. those quantified in this study) and discuss the implications of the suggested circular strategies.

Practical implications of this research may primarily pertain to reuse and tailored use of devices, even if telemonitoring was implemented previously. Whereas several reasons can exist to use telemonitoring in clinical follow-up, we must balance potential benefits with incurred resource use and environmental repercussions. Notwithstanding our finding that patients supported sustainable use strategies, they should be consulted locally for their willingness to collaborate.

To conclude, telemonitoring follow-up can have a substantially larger environmental impact than CoS, mainly due to the production of required devices. Patient commute distance, reuse of devices, and tailored use of devices should be considered when using telemonitoring for clinical follow-up—preferably early during implementation. Healthcare professionals and patients generally seem supportive of sustainable healthcare, yet may not consider it in their choice to use telemonitoring. Rather, a default option which uses telemonitoring in the most environmentally sustainable way avoids the need to discuss it during clinical consultations. With the right attention, healthcare that benefits both the patient and the planet is possible.

## Supplementary Material

ztaf012_Supplementary_Data

## Data Availability

As much data as possible related to the life cycle inventory and modelling choices has been made available in the manuscript and supplementary files to allow for more detailed appreciation of the environmental impact assessment, quality control, and further use of the study findings. Moreover, study findings will be uploaded to the open access research database healthcarelca.com in due time. Any further information, including anonymized interview transcripts and data matrices, can be obtained from the authors upon reasonable request within a reasonable timeframe. An exception applies to additional (manufacturer) reporting of the environmental impact of eHealth devices, for which a non-disclosure agreement had to be signed.

## Lead author biography

Egid van Bree is a medical doctor and PhD-candidate with a special interest in environmental sustainability in healthcare. Throughout his research, he focusses on the quantification of the environmental impact of care pathways, the clarification of patient and healthcare professional perspectives on environmentally sustainable healthcare, and the implementation of evidence-based initiatives to green clinical practice. Next to his daily work, he is a board member of the Dutch Green Health Alliance (Groene Zorg Alliantie): a bottom-up network of workplace initiatives striving for environmentally sustainable healthcare and planetary health.
