# Supplementary material for: The environmental impact of telemonitoring vs. on-site cardiac follow-up: a mixed-method study
Source: Eur Heart J Digit Health. 2025 Feb 26;6(3):496–507. doi: 10.1093/ehjdh/ztaf012 (PMC12088715; doi:10.1093/ehjdh/ztaf012)
Supplement: ztaf012_Supplementary_Data [file ztaf012_supplementary_data.pdf]

Supplementary material to

The environmental impact of telemonitoring versus on-site cardiac follow-up: a mixed-method study

Table of contents

Supplement A – Transparency checklist for quantifying greenhouse gas emissions of telemedicine page 2

Supplement B – Life cycle inventory and modelling page 3–13

Supplement C – Topic list interviews and focus groups page 14–17

Supplement D – Life cycle impact assessment and sensitivity/uncertainty analysis for care on site and telemonitoring page 18–28

References page 29

Supplement A – Transparency checklist for quantifying greenhouse gas emissions of telemedicine

Item	Criterion	Relevant information
1	Does the study specify its aim?	Introduction section, last paragraph
2	Does the study specify its functional unit?	Methods section, 'goal and scope'
3	Does the study specify its reference flow?	Specified in methods section, 'follow-up protocol', Figure 1, and Supplement B
4	Does the study provide a description of the life cycle stages?	Where applicable, life cycle stages are mentioned in the methods section 'goal and scope', Figure 1, and Supplement B
5	Does the study provide a list of important unit processes?	Information regarding included processes provided in Supplement B, statement regarding availability at the bottom of Supplement B
6	Does the study specify exclusions and reasons for exclusions?	The study was based on a preceding clinical trial, including patients with ST-segment elevation MI or non-ST-segment acute coronary syndrome. As reported in the preceding clinical trial (CONSORT flowchart), 75 patients were excluded from participation because they declined to participate in the trial.
7	Does the study specify the system boundary?	Specified in methods section 'goal and scope', Figure 1, and Supplement B
8	Does the study provide the source for all data used in the analysis?	Detailed specification in Supplement B
9	Does the study assess the temporal representativeness of the data?	Reference years of used data are included in Supplement B. For the telemonitoring devices (smartwatch, blood pressure monitor, and body weight scale) we used own disassembly data of the devices in use and most recent available manufacturer data. For other processes, the most recent data were used to optimize temporal representativeness based on availability (e.g. for Power Use Effectiveness (PUE) of data transfer). Representativeness was also included in the uncertainty analysis for the reference scenario by using pedigree matrix-computed uncertainty ranges.
10	Does the study assess the geographical representativeness of the data?	Where possible, the most geographically representative data were used. This relates most to the sources of energy generation and types of vehicles used – for which we used datasets tailored to the Netherlands. Representativeness was also included in the uncertainty analysis for the reference scenario by using pedigree matrix-computed uncertainty ranges.
11	Does the study assess the technological representativeness of the data?	Where possible, the most technologically representative data were used. This relates mostly to the telemonitoring devices (smartwatch, blood pressure monitor, and body weight scale), the PUE, and the production of SSD storage. Representativeness was also included in the uncertainty analysis for the reference scenario by using pedigree matrix-computed uncertainty ranges.
12	Does the study assess the completeness of the data?	Statements regarding completeness of foreground and background data were included in Supplement B. During the research, EvB and LS assessed the completeness of the dataset at several timepoints and added information (e.g. the direct emissions due to combustion of natural gas) when available.
13	Does the study estimate the carbon footprint in terms of CO2eq?	Methods section, 'data analysis'
14	Does the study provide a list of greenhouse gasses taken into account?	Where information is available, the datasets in the study contain information regarding multiple greenhouse gases (carbon dioxide, methane, nitrous oxide, PCFs, HCFs, and sulphur hexafluoride)
15	Does the study specify the selected characterization factors?	Methods section, 'data analysis'. Characterization factors are included in the Environmental Footprint method v3.1 (based on scientific consensus). Kindly refer to the guiding documents of the Joint Research Centre (JRC) of the European Commission for further information.
16	Does the study report the selected allocation procedures?	Methods section, 'Table 1', Discussion section 'limitations', and Supplement B
17	Does the study report the outcomes per unit of analysis?	The results were reported according to the functional unit
18	Does the study report the carbon footprint separately per specific component?	Results section: 'Table 2', 'Figure 2', and 'environmental impact'. Supplement D for further details.
19	Does the study report the carbon footprint according to life cycle phases?	We reported environmental impacts based on elements of care included in the reference flow. We included a statement regarding the life cycle contribution of the tablets in the Results section, 'environmental impact'
20	Does the study report a qualitative statement on the influence of key uncertainties or methodological choices on the result?	Results section: 'use strategies', 'commute distance', and 'database choices'; Discussion section; and Supplement D
21	Does the study perform a quantitative sensitivity analysis?	Results section, 'use strategies' and 'commute distances'; Supplement D
22	Does the study critically discuss limitations, e.g., appropriateness of system boundary, data quality, or methods of analysis?	Limitations regarding foreground data are discussed in the Discussion section, (more specifically: limitations and contextualization); more detailed statements regarding specific life cycle inventory elements are included in Supplement B (e.g. data transfer / storage / computing)

Note to reader: this table of assessment criteria is based on Table 1 of the article *A Transparency Checklist for Carbon Footprint Calculations Applied within a Systematic Review of Virtual Care Interventions* by Lange et al (2022), doi: 10.3390/ijerph19127474

Supplement B – Life cycle inventory and modelling

Table B1. Life cycle inventory overview

Process category	Subcategory	Data collection	Data modelling LCI
eHealth devices	Smartwatch	Information regarding the environmental impact of production, distribution, usage, and disposal of the exact smartwatch in use was obtained from the manufacturer (ref year 2022). Reporting is currently protected by a confidentiality agreement and was therefore not included in this appendix.<br><br>As an alternative, we completely disassembled a smartwatch, studied its materials, and weighed components individually using a precision scale (0.01g). This process ("own data") is included in this appendix.	The manufacturer information (calculated using the EF 3.0 method and reported in global warming, fine particulate matter, fossil resource use, mineral and metal resource use, and water use) was added separately outside of SimaPro. Calculated numbers can be found in the results appendix.<br><br>The "own data" version was based on own modelling using ecoinvent market processes. Since exact material compositions were unknown, some electronic components were too small to disassemble (e.g. "electronic hour parts"), and since some processes were unavailable in the ecoinvent database (e.g. "Li-polymer battery" or "fluoroelastomere wristband", meaning that proxy processes had to be used); the modelled 'own data' should be interpreted as an approximation.<br><br>To investigate differences, we compared and reported (see results appendix) manufacturer outcomes and own calculations for environmental impact of the smartwatch.
	Body weight scale	Information regarding the environmental impact of production, distribution, usage, and disposal of the body weight scale in use was obtained from the manufacturer (ref year 2022). Reporting is currently protected by a confidentiality agreement and was therefore not included in this appendix.<br><br>As an alternative, we completely disassembled a body weight scale, studied its materials, and weighed components individually using a precision scale (0.01g). This process ("own data") is included in this appendix.	The manufacturer information (calculated using the EF 3.0 method and reported in global warming, fine particulate matter, fossil resource use, mineral and metal resource use, and water use) was added separately outside of SimaPro. Calculated numbers can be found in the results appendix.<br><br>The "own data" version was based on own modelling using ecoinvent market processes. Since exact material compositions were unknown, some electronic components were too small to disassemble (e.g. "minor LED-strip/buttons at bottom of LCD"), and since some processes were unavailable in the ecoinvent database (e.g. the specific type of ABS plastic or other hard plastic, meaning that proxy processes had to be used); the modelled 'own data' should be interpreted as an approximation.<br><br>To investigate differences, we compared and reported (see results appendix) manufacturer outcomes and own calculations for the environmental impact of the body weight scale.
	Blood pressure monitor (BPM)	Information regarding environmental impact of the blood pressure monitor in use was obtained from the manufacturer (ref year 2022). Reporting is currently protected by a confidentiality agreement and was therefore not included in this appendix.<br><br>As an alternative, we completely disassembled a blood pressure monitor, studied its materials, and weighed components individually using a precision scale (0.01g). This process ("own data") is included in this appendix.	The manufacturer information (reported in global warming only) was compared separately, outside of SimaPro.<br><br>The "own data" version was based on own modelling using ecoinvent market processes. Since exact material compositions were unknown, some electronic components were too small to disassemble (e.g. "PCB containing LEDs" and the "air pump"), and since some processes were unavailable in the ecoinvent database (e.g. "Li-polymer battery" or "air compressor", meaning that proxy processes had to be used); the modelled 'own data' should be interpreted as an approximation.<br><br>We used our own calculations for the

			environmental impact of the BPM in the complete analysis.
Commute	Patient commute	Average distance based on preceding study of telemonitoring intervention by Treskes et al (2022)[1] and verified by interviews with nurse practitioners. Means of travel based on interviews with nurse practitioners.	The transportation processes tailored to the Dutch market for transportation of goods (Goederenvervoer STREAM 2020; CE Delft, 2021)[2] and commute of persons (Personenvervoer STREAM 2022; CE Delft, 2023)[3] were used as market processes (S/U), adjusted from the standard cut-off by classification market processes in ecoinvent v3.9.1 for the same fuel types and vehicle types. The adjusted processes were previously commissioned by the Dutch Government. Adjustments are made for the processes that represent the production of fuels, such as gasoline (i.e. Well-To-Tank emissions), e.g. gasoline currently contains 10% biofuels, rather than 5% biofuels, resulting in a lower requirement of crude oil and higher requirement of land use for production of biofuels. And adjustments are made for the processes that represent the combustion of fuels and driving of vehicles (i.e. Tank-To-Wheel emissions), e.g. the amount of PM2.5 that is emitted during the process of driving a car, based on direct measurements of Dutch vehicles.<br><br>Effectively, this results in: 1) replacement of the CO2eq, NOx, SO2, NMVOC, and PM2.5 emissions documented in ecoinvent processes by the vehicle measurements conducted in the Netherlands; 2) for commute of persons the reporting unit of cars and motorbikes was adjusted based on the average occupation of vehicles ('km' to 'personkm'), such as a factor 1.31 for the average car. For further details/reporting, kindly consult the authors.<br><br>Modelled travel distance in a process representing the 'average' individual patient over the course of one year post-MI follow-up. Average percentages for means of transport (e.g. 50% car transport) were used to allocate 50% of the travel distance by car and the remaining 50% by bike or public transport. Refer to process tab for details.
	Employee commute	Average distance and means of travel based on hospital-wide internal audit (2020) of employee commute during day and night time.<br><br>Number of appointments and staff involved - including their workload for allocation - based on protocol analysis, interviews with nurse practitioners, and own assumptions based on clinical experience.	Explanation of transportation process as explained above for "patient commute"<br><br>Own modelling of travel distance for all staff involved over the course of a one year post-MI follow-up. Allocation of travel based on allocation of staff workload to the follow-up of a single patient. Modelled travel distance in a process representing the 'average' hospital employee, based on the internal audit. Average percentages (e.g. 21.8% car travel) were used to allocate 21.8% of the travel distance by car and the remaining percentage by bike or public transport. Refer to process tab for details.
Diagnostic tests	Cardiac ultrasound	Number of ultrasounds based on protocol analysis and interviews with nurse practitioners. Required materials based on previous research in other Dutch academic hospital and own assumptions based on clinical experience.	Own modelling using ecoinvent market processes. Ultrasound energy use based on McAlister et al (2022)[4] study; ultrasound gel, nitrile glove, laundering process towel based on previous modelling in other Dutch academic hospital.
	Blood tests	Number of blood tests based on protocol analysis and interviews with nurse practitioners. Required materials based on previous research in other Dutch academic	Own modelling using ecoinvent market processes. Material and energy use based on McAlister et al (2021)[5] pathology testing study, complemented by phlebotomy as described in Spoyalo et al (2023)[6] study.

		hospital and own assumptions based on clinical experience.	Spoyalo study additionally modelled for comparison of results.
	Electrocardiogram (ECG)	Number of ECGs based on protocol analysis and interviews with nurse practitioners. Required materials based on previous research in other Dutch academic hospital and own assumptions based on clinical experience.	Own modelling using ecoinvent market processes. Material use largely based on previous modelling in other Dutch academic hospital, including ultrasound gel, nitrile glove, and ECG cables. Complemented by available manufacturer data on device energy use.
	Holter monitoring (Holter)	Number of Holters based on protocol analysis and interviews with nurse practitioners. Required materials and means of usage based on interviews with cardiology support staff responsible for application, distribution, and return of Holter devices.	Own modelling using ecoinvent market processes. Device production not included due to frequent usage (minimal impact after allocation to single patient). ECG cables based on previous modelling in other Dutch academic hospital. Alkaline battery based on MIT LCA (2011)[7] appendix. Partially included transportation of Holter via email.
On site energy + materials	Building energy use	Average energy use for outpatient clinic based on annual energy reporting of the hospital (2022). Duration and workload for patient consultations based on interviews with nurse practitioners.	The average electricity mix tailored to the Dutch market in 2021 "Elektriciteit gemiddeld - NL 2021" is a market process (S) that was created by CE Delft (Delft, the Netherlands) in 2023-2024 [8], commissioned by the Dutch Government and based on energy generation data as collected by the Dutch National Institute for Statistics (CBS) and National Planning Bureau for the Environment (PBL).<br><br>Converted to energy use per day, allocated to consultation of a single patient based on (assumed) size of outpatient clinic rooms. Further allocated to individual patient based on average workload (no. of patients and admin work in a single outpatient clinic room) and duration of consultation. Separately added incineration of natural gas.
	Hand disinfectant	Usage based on interviews with nurse practitioners. Amount and relevant products based on previous publication (Thiel et al 2023)[9] and previous research in other Dutch academic hospital.	Own modelling using ecoinvent market processes.
	Examination table paper	Usage based on own assumptions based on clinical experience.	Own modelling using ecoinvent market processes.
	Computer use (in consultation room)	Usage based on own assumptions based on clinical experience.	Own modelling using adjusted ecoinvent market process. Excluded energy use of computer to avoid double counting (since energy use is already included in the Building Energy Use process). Compared impact of computer usage to impact of computers as calculated for other Dutch academic hospital with similar circumstances (in description).
Digital infrastructure	Data transfer / storage / computing	Required amount of data, storage sites, location of storage, and number of data transfers based on interviews with IT-specialists at academic hospital. Required amount of energy for data transfer and cloud storage based on consultation of knowledge management/IT experts and existing publications with a recent overview of Power Use Effectiveness (PUEs) (Jackson et al 2023, Swiss Federal Office of Energy 2022). [10,11]<br><br>Information regarding production of SSD storage based on publicly available LCA information of SSD-manufacturer.	Explanation of electricity mix as stated in "Building energy use".<br><br>Modelled data transfer and online storage (based on total one year follow-up duration) for calculated data size (in GB), including the 'Jackson' and 'Swiss' process for the PUEs where possible and comparing them in sensitivity analysis. Based on geolocation of online storage, chose the German of French electricity mix as available in ecoinvent accordingly. Due to limited data availability, only the electricity use is included in the model. Water use for cooling is not included.<br><br>Environmental impact of SSD production (based on required data storage size, in GB) was entered directly as environmental impact based on Seagate (2016) [12] LCA reporting. Added Tannu et al (2022) [13] review for recent comparison - only available for carbon footprint of SSD production.

	Laptop use (by patient)	Usage based on own assumption.	Own modelling using ecoinvent market processes.
	Video calling	Duration based on interviews with nurse practitioners. Required amount of data based on average of multiple online sources (gadgetshouse.com / businesstechplanet.com).	Explanation of electricity mix as stated in "Building energy use". Explanation of PUE modelling for data transfer (required energy to transfer average data size) as stated in "Data transfer / storage / computing".
NB: kindly note that the majority of subcategories listed above have been included as separate tabs in an excel sheet for further details (and transparency) of the modelling process.			
NB2: for all disposables, plastic- and metal specific incineration processes were modelled; if specific plastics were unavailable or if disposables consisted of other materials, the municipal incineration was chosen.			
NB3: for eHealth devices, the 'electronic waste' processes available in ecoinvent were chosen. Considering that information was unavailable regarding disposal of the devices (happens by patients themselves).			

Table B2. Modelling of comparison Care on Site and Telemonitoring

**SimaPro 9.5.0.1**	**Project: LCA eHealth**		
**Process**	**Amount**	**Unit**	**Remarks**
1. eHealth (telemonitoring) follow-up MI	1	p	
**Includes:**			
Transport - allocation of staff commute for eHealth follow-up (Box)	1	p	Transport allocation of staff commute. Includes nurse physician (4x), TTE (2x), Holter (1x), ECG (2x). Allocated based on workload - refer to process for further details.
Transport - outpatient average [distance conditional; assumed C50B25OV25]	2	p	In regular MI patient follow-up, a patient visits the hospital 2 times (3 and 12 months). Process includes transportation back-and-forth. Assumed 50% transportation by car based on interview with nurse, possible scenario 100% transportation by car.
Hospital visit - cardiac ultrasound (TTE)	2	p	1 TTE is performed at 3 months follow-up and 1 at 12 months follow-up. Process includes energy and material required. No additional transportation (simultaneous with follow-up appointment)
Hospital visit - Holter	1	p	1 Holter is performed at 3 months follow-up. Process includes material required. No additional transportation for pick-up (simultaneous with follow-up appointment) or drop-off (simultaneous with revalidation appointment - outside of scope).
Hospital visit - examination table paper	1*0,2*2	p	Physical examination 2/10 patients (20% allocation, based on interview nurse physician); in total 2 follow-up visits (2x).
Hospital visit - hand disinfection, single hand disinfection	2*2	p	Hand disinfection before/during and after physicial consultation, based on interview with nurse physician. 2* disinfection, 2* physical visit
Hospital visit - ECG recording (single, 12 lead)	2	p	ECG recording performed at every physical visit. Includes material and energy usage.
Hospital visit - regular blood tests [method conditional]	1,45	p	Lab test performed 1 time, and +1 time in 40-50% of cases (estimation by nurse physician). Therefore *1,45. Materials and energy use included.
Energy - outpatient clinic energy usage [adjust allocation] [energy conditional]	4	p	Energy required for outpatient clinic room (light, computers, HVAC), allocated per patient visit (review process for more details). In total 2 physical visits and 2 online visits, conducted in the same room (4x).
the Box - MI follow-up, proxies [device condition]	1	p	Proxy process for the contents of 'the Box' including smartwatch, smart scale, and blood pressure monitor. Allocated 100% to follow-up period, considering they are given to the patient after follow-up is completed.
the Box - digital infrastructure MI-follow-up	1	p	Process for the data transfer and storage required while using 'the Box'. Includes device manufacturer, local storage (3x), and cloud storage.
the Box - video call (15 min) MI-follow-up	2	p	Process for the data transfer required while video calling in the MI-follow-up process using the Box. Process represents a single video call of 15 minutes using Microsoft Teams.

Hospital visit - computer usage, per hour [Ecoinvent EU proxy, 2005]	4*consultation_dura tion+patient_support _time	p	Process accounting for staff computer usage, including standby/off-time. Considering 4 * 15min consultation. Assumed an extra 1h of computer time for patient support during the 1y follow-up.
eHealth - laptop usage, per hour [Ecoinvent proxy 2005]	2*consultation_dura tion	p	Process accounting for patient laptop computer usage (no phone or tablet available in ecoinvent), only considering 'on-time' as the device is assumed to be used for a many other purposes than the 2 x 15min consultation.
**Based on:**			

**Input parameters**	**Amount**	**Distribution**	**Lower**	**Upper**	**Remarks**
consultation_duration	0,25	Triangle	0,125	0,3333	Average duration assumed 15mins per patient, included variation of 7,5min per patient (factor 0,125) or extended duration of 20mins per patient (factor 0,3333)
patient_support_time	1	Uniform	0,5	1,5	Assumed average of 1h extra computer support time during entire 1y follow-up, included variation of 30mins to 1,5h

**SimaPro 9.5.0.1**	**Project: LCA eHealth**		
**Process**	**Amount**	**Unit**	**Remarks**
1. Physical follow-up MI	1	p	
Includes:			
Transport - allocation of staff commute for physical follow-up	1	p	Transport allocation of staff commute. Includes nurse physician (4x), TTE (1x), Holter (1x), ECG (4x). Allocated based on workload - refer to process for further details.
Transport - outpatient average [distance conditional; assumed C50B25OV25]	4	p	In regular MI patient follow-up, a patient visits the hospital 4 times (1, 3, 6, and 12 months). Process includes transportation back-and-forth. Assumed 50% transportation by car based on interview with nurse, possible scenario 100% transportation by car.
Hospital visit - cardiac ultrasound (TTE)	2	p	1 TTE is performed at 3 months follow-up and 1 at 12 months follow-up. Process includes energy and material required. No additional transportation (simultaneous with follow-up appointment)
Hospital visit - Holter	1	p	1 Holter is performed at 3 months follow-up. Process includes material required. No additional transportation for pick-up (simultaneous with follow-up appointment) or drop-off (simultaneous with revalidation appointment - outside of scope).
Hospital visit - examination table paper	1*0,2*4	p	Physical examination 2/10 patients (20% allocation, based on interview nurse physician); in total 4 follow-up visits (4x).
Hospital visit - hand disinfection, single hand disinfection	2*4	p	Hand disinfection before/during and after physical consultation, based on interview nurse physician; 2* per visit, in total 4 follow-up visits
Hospital visit - ECG recording (single, 12 lead)	4	p	ECG recording performed at every physical visit. Includes material and energy usage.
Hospital visit - regular blood tests [method conditional]	2,45	p	Lab test performed 2 times, and +1 time in 40-50% of cases (estimation by nurse physician). Therefore *2,45. Materials and energy use included.
Energy - outpatient clinic energy usage [adjust allocation] [energy conditional]	4	p	Energy required for outpatient clinic room (light, computers, HVAC), allocated per patient visit (review process for more details). In total 4 physical visits (4x).
Hospital visit - computer usage, per hour [Ecoinvent EU proxy, 2005]	4*consultation_dura tion+patient_support _time	p	Process accounting for staff computer usage, including standby/off-time. Considering 4 * 15min consultation. Assumed an extra 1h of computer time for patient support during the 1y follow-up.
**Based on:**			

**Input parameters**	**Amount**	**Distribution**	**Lower**	**Upper**	**Remarks**
consultation_duration	0,25	Triangle	0,125	0,3333	Average duration assumed 15mins per patient, included variation of 7,5min per patient (factor 0,125) or extended duration of 20mins per patient (factor 0,3333)
patient_support_time	1	Uniform	0,5	1,5	Assumed average of 1h extra computer support time during entire 1y follow-up, included variation of 30mins to 1,5h

Project Input parameters	Amount	Distribution	Lower	Upper	Remarks
patient_transport	14	Lognormal (1)	n/a	n/a	Average distance of LUMC cardiology MI follow-up patient to hospital (=7km *2); chosen a lognormal distribution assuming that average travel distance would be a right-skewed distribution
employee_transport	35,6	Lognormal (1)	n/a	n/a	Average distance of LUMC employees home to work (=17,8km *2); chosen a lognormal distribution assuming that average travel distance would be a right-skewed distribution
number_patients	25	Triangle	15	30	Allocation factor for the energy usage of outpatient consultation rooms (divided by). Based on the total number of patients seen by one healthcare professional in the outpatient clinic in one day (=20; 15 mins per patient), and assuming that the same room is used for administrative work for an additional 1h 15 mins (=+5 patients).

Table B3. Process "Smartwatch (own data)"

Product	Amount	Unit	Allocation
eHealth - smartwatch [own data]	1	p	100
**Resources**	**Amount**	**Unit**	**Remarks**
**Materials/fuels**			
Liquid crystal display, unmounted, mobile device {GLO}| market for liquid crystal display, unmounted, mobile device | Cut-off, S	0,68	g	LCD
Printed wiring board, surface mounted, unspecified, Pb free {GLO}| market for printed wiring board, surface mounted, unspecified, Pb free | Cut-off, S	2,9	g	PCB
Steel, chromium steel 18/8 {GLO}| market for steel, chromium steel 18/8 | Cut-off, S	11,8	g	main case steel
Steel, chromium steel 18/8 {GLO}| market for steel, chromium steel 18/8 | Cut-off, S	1,23	g	hourglass ring steel, weighed minus 4.9g glass
Flat glass, coated {RER}| market for flat glass, coated | Cut-off, S	4,9	g	hourglass
Steel, chromium steel 18/8 {GLO}| market for steel, chromium steel 18/8 | Cut-off, S	4,17	g	bottom of watch steel, weighed, minus assumed 1.5g glass
Flat glass, coated {RER}| market for flat glass, coated | Cut-off, S	1,5	g	bottom glass, weight assumed
Battery, Li-ion, LiMn2O4, rechargeable, prismatic {GLO}| market for battery, Li-ion, LiMn2O4, rechargeable, prismatic | Cut-off, S	2,52	g	in reality Li-polymer battery, not available in Ecoinvent therefore chosen this rechargeable battery

Acrylonitrile-butadiene-styrene copolymer {GLO}| market for acrylonitrile-butadiene-styrene copolymer | Cut-off, S	0,76	g	Plastic inside watch case
Aluminium, primary, ingot {IAI Area, EU27 & EFTA}| market for aluminium, primary, ingot | Cut-off, S	2,49	g	hourplate
Aluminium, primary, ingot {IAI Area, EU27 & EFTA}| market for aluminium, primary, ingot | Cut-off, S	1,41	g	PCB cover with yellow-golden coating
Electronic component, active, unspecified {GLO}| market for electronic component, active, unspecified | Cut-off, S	1,024	g	electronic hour parts (3x)
Brass {RoW}| market for brass | Cut-off, S	0,008	g	coating of analog dial
Chromium {GLO}| market for chromium | Cut-off, S	0,01	g	coating of hands
Synthetic rubber {GLO}| market for synthetic rubber | Cut-off, S	15,65	g	wristbands ('fluoroelastomere' not available)
Steel, chromium steel 18/8 {GLO}| market for steel, chromium steel 18/8 | Cut-off, S	3,23	g	buckle of rubber wristband
Steel, chromium steel 18/8 {GLO}| market for steel, chromium steel 18/8 | Cut-off, S	0,18	g	fixture of rubber band (x2)
Printed wiring board, surface mounted, unspecified, Pb free {GLO}| market for printed wiring board, surface mounted, unspecified, Pb free | Cut-off, S	0,024	g	mini PCB-like components
Brass {RoW}| market for brass | Cut-off, S	0,04	g	small brass/gold-coloured piece
**Electricity/heat**			
Electricity, low voltage {RU}| market for electricity, low voltage | Cut-off, S	15,5	kWh	Energy for tempering and shaping of hourglass, based on manufacturer information, produced in Russia
Metal working, average for chromium steel product manufacturing {GLO}| market for metal working, average for chromium steel product manufacturing | Cut-off, S	11,8	g	main case steel
Metal working, average for chromium steel product manufacturing {GLO}| market for metal working, average for chromium steel product manufacturing | Cut-off, S	1,23	g	steel ring hourglass
Metal working, average for chromium steel product manufacturing {GLO}| market for metal working, average for chromium steel product manufacturing | Cut-off, S	4,17	g	steel bottom
Injection moulding {GLO}| market for injection moulding | Cut-off, S	0,76	g	plastic inside watch case
Metal working, average for aluminium product manufacturing {GLO}| market for metal working, average for aluminium product manufacturing | Cut-off, S	2,49	g	hourplate
Metal working, average for aluminium product manufacturing {GLO}| market for metal working, average for	1,41	g	PCB cover

aluminium product manufacturing | Cut-off, S			
Metal working, average for metal product manufacturing {GLO}| market for metal working, average for metal product manufacturing | Cut-off, S	0,018	g	analog dial and hands
Injection moulding {GLO}| market for injection moulding | Cut-off, S	15,65	g	wristbands
Metal working, average for chromium steel product manufacturing {GLO}| market for metal working, average for chromium steel product manufacturing | Cut-off, S	3,41	g	buckle and fixtures (2x)
Transport, Road, Bulk/piece, Lorry medium weight - STREAM	27,293	kgkm	Transport finished product Paris to Leiden (approx 500km) multiplied by total weight product = 54,586g excl packaging
Transport, Road, Bulk/piece, Lorry medium weight - STREAM	109,172	kgkm	Assumed transport intermediate components Le Havre port to Paris (approx 200). Total weight product = 54,586g excl packaging. Multiplied by factor 10 to compensate for indirect transport via warehouses or intermediary facilitaties
Transport, Shipping, Bulk/piece, Deep sea: Bulk carrier 35-60 dwkt - STREAM	10371,34	kgkm	Assumed transport intermediate components Shanghai to Le Havre (approx 19k km). Total weight product = 54,586g excl packaging. Multiplied by factor 10 to compensate for indirect transport via warehouses or intermediary production sites.
**Waste to treatment**			
Used Li-ion battery {GLO}| market for used Li-ion battery | Cut-off, S	2,52	g	used as proxy for battery treatment
Used smartphone {GLO}| market for used smartphone | Cut-off, S	52,066	g	used as proxy for electronic waste treatment (since device in question is a high tech device)

Table B4. Process "Body weight scale (own data)"

**Product**	**Amount**	**Unit**	**Allocation**
eHealth - body weight scale [own data]	1	p	100
**Resources**	**Amount**	**Unit**	**Remarks**
**Materials/fuels**			
Flat glass, coated {RER}| market for flat glass, coated | Cut-off, S	1,555	kg	Actual glass is coated with ITO (Indium Tin Oxide) rather than Nickel-Chromium/Bismuth (ecoinvent process)
Indium tin oxide powder, nanoscale, for sputtering target {RER}| market for indium tin oxide powder, nanoscale, for sputtering target | Cut-off, S	0,0000597	kg	Own addition of ITO based on weight of Nickel and Chromium in Ecoinvent glass coating process.
Steel, low-alloyed {GLO}| market for steel, low-alloyed | Cut-off, S	4	g	Large screws (10x)
Steel, low-alloyed {GLO}| market for steel, low-alloyed | Cut-off, S	0,12	g	Small screws (2x)
Printed wiring board, surface mounted, unspecified, Pb free {GLO}| market for printed wiring board, surface mounted, unspecified, Pb free | Cut-off, S	10,4	g	
Liquid crystal display, unmounted {GLO}| market for liquid crystal display, unmounted | Cut-off, S	20	g	
Polycarbonate {GLO}| market for polycarbonate | Cut-off, S	17,6	g	Carpet feet outside scale
Acrylonitrile-butadiene-styrene copolymer {GLO}| market for acrylonitrile-butadiene-styrene copolymer | Cut-off, S	8,54	g	Feet inside scale, partially metal (assumed 1/3) and partially plastic (assumed 2/3, assumed ABS); 4x feet
Steel, low-alloyed {GLO}| market for steel, low-alloyed | Cut-off, S	4,26	g	Feet inside scale, partially metal (assumed 1/3) and partially plastic (assumed 2/3, assumed ABS); 4x feet
Acrylonitrile-butadiene-styrene copolymer {GLO}| market for acrylonitrile-butadiene-styrene copolymer | Cut-off, S	20	g	Inside feet cover, partially hard plastic (2/3, assumed ABS) and partially foam (1/3, assumed PUR)

Polyurethane adhesive {GLO}| market for polyurethane adhesive | Cut-off, S	10	g	Inside feet cover, partially hard plastic (2/3, assumed ABS) and partially foam (1/3, assumed PUR)
Polyethylene, high density, granulate {GLO}| market for polyethylene, high density, granulate | Cut-off, S	0,6	g	Plastic cover of LCD screen, assumed HDPE
Cable, unspecified {GLO}| market for cable, unspecified | Cut-off, S	4,8	g	wires/cables connecting electricity
Steel, low-alloyed {GLO}| market for steel, low-alloyed | Cut-off, S	43,2	g	metal sensor inside foot, 4x
Electronic component, active, unspecified {GLO}| market for electronic component, active, unspecified | Cut-off, S	0,26	g	minor LED-strip/buttons at bottom of LCD
Acrylonitrile-butadiene-styrene copolymer {GLO}| market for acrylonitrile-butadiene-styrene copolymer | Cut-off, S	6,4	g	plastic case of LCD
Polyurethane adhesive {GLO}| market for polyurethane adhesive | Cut-off, S	0,6	g	foam around LCD, adhesive
Polycarbonate {GLO}| market for polycarbonate | Cut-off, S	303,2	g	outer plastic case/bottom of scale
Energy - Alkaline battery, disposable [adjust allocation] [excludes transport]	1,35	p	Adjusted for 4 AAA batteries of 11g (process modeled for large AA battery of 32.5g)
**Electricity/heat**			
Metal working, average for steel product manufacturing {GLO}| market for metal working, average for steel product manufacturing | Cut-off, S	4	g	Large screws
Metal working, average for steel product manufacturing {GLO}| market for metal working, average for steel product manufacturing | Cut-off, S	0,12	g	Small screws
Injection moulding {GLO}| market for injection moulding | Cut-off, S	17,6	g	Carpet feet
Injection moulding {GLO}| market for injection moulding | Cut-off, S	8,54	g	Feet inside scale
Metal working, average for steel product manufacturing {GLO}| market for metal working, average for steel product manufacturing | Cut-off, S	4,26	g	Feet inside scale
Injection moulding {GLO}| market for injection moulding | Cut-off, S	20	g	Inside feet cover
Extrusion of plastic sheets and thermoforming, inline {GLO}| market for extrusion of plastic sheets and thermoforming, inline | Cut-off, S	0,6	g	Plastic cover LCD screen
Metal working, average for steel product manufacturing {GLO}| market for metal working, average for steel product manufacturing | Cut-off, S	43,2	g	metal sensor inside foot
Injection moulding {GLO}| market for injection moulding | Cut-off, S	6,4	g	plastic case of LCD
Injection moulding {GLO}| market for injection moulding | Cut-off, S	303,2	g	outer plastic case of scale
Electricity, low voltage {Europe without Switzerland}| market group for electricity, low voltage | Cut-off, S	2,3	kWh	"transformation of glass components", based on company information, production site unknown
Transport, Road, Bulk/piece, Lorry medium weight - STREAM	1030	kgkm	Transport finished product Paris to Leiden (approx 500km) multiplied by total weight product = 2.053kg excl packaging
Transport, Road, Bulk/piece, Lorry medium weight - STREAM	4110	kgkm	Assumed transport intermediate components Le Havre port to Paris (approx 200). Total weight product = 2.053kg excl packaging. Multiplied by factor 10 to compensate for indirect transport via warehouses or intermediary processing sites.
Transport, Shipping, Bulk/piece, Deep sea: Bulk carrier 35-60 dwkt - STREAM	390000	kgkm	Assumed transport intermediate components Shanghai to Le Havre (approx 19k km). Total weight product = 2.053kg excl packaging. Multiplied by factor 10 to compensate for indirect transport via warehouses or intermediary production facilities.
Tempering, flat glass {GLO}| market for tempering, flat glass | Cut-off, S	1,555	kg	Added for glass plate
**Waste to treatment**			
Waste electric and electronic equipment {GLO}| market for waste electric and electronic equipment | Cut-off, S	0,498	kg	Other waste
Waste glass {NL}| market for waste glass | Cut-off, S	1,555	kg	Glass plate

Table B5. Process "Blood pressure monitor (own data)"

Product	Amount	Unit	Allocation
eHealth - blood pressure monitor [own data]	1	p	100
**Resources**	**Amount**	**Unit**	**Remarks**
**Materials/fuels**			
Textile, nonwoven polyester {GLO}| market for textile, nonwoven polyester | Cut-off, S	56,5	g	Manchet (band that goed around upper arm)
Polyethylene, low density, granulate {GLO}| market for polyethylene, low density, granulate | Cut-off, S	21	g	Inflatable balloon inside manchet that exercises pressure on arm/vessel
Steel, chromium steel 18/8 {GLO}| market for steel, chromium steel 18/8 | Cut-off, S	30,6	g	Metal oval ring, part of manchet
Polycarbonate {GLO}| market for polycarbonate | Cut-off, S	23,6	g	Plastic case of BPM, upper side, hard plastics only
Polycarbonate {GLO}| market for polycarbonate | Cut-off, S	32,2	g	Plastic case of BPM, bottom side, some mixed plastics on the inner side
Polyethylene, high density, granulate {GLO}| market for polyethylene, high density, granulate | Cut-off, S	9	g	Plastic attachment of inflatable balloon
Printed wiring board, surface mounted, unspecified, Pb free {GLO}| market for printed wiring board, surface mounted, unspecified, Pb free | Cut-off, S	13,4	g	PCB (NB: actual PCB also contains LEDs, which were not added separately)
Synthetic rubber {GLO}| market for synthetic rubber | Cut-off, S	1,4	g	Rubber seal of LEDs
Polyethylene, high density, granulate {GLO}| market for polyethylene, high density, granulate | Cut-off, S	0,4	g	Plastic button
Aluminium, primary, ingot {IAI Area, EU27 & EFTA}| market for aluminium, primary, ingot | Cut-off, S	0,1	g	Aluminium ring
Polyvinylchloride, bulk polymerised {GLO}| market for polyvinylchloride, bulk polymerised | Cut-off, S	1,6	g	Tubing for air, connected to balloon and air vent
Battery, Li-ion, LiMn2O4, rechargeable, prismatic {GLO}| market for battery, Li-ion, LiMn2O4, rechargeable, prismatic | Cut-off, S	19	g	Lithium polymer battery
Permanent magnet, for electric motor {GLO}| market for permanent magnet, for electric motor | Cut-off, S	10	g	Magnets in manchet (NB: only magnet dataset available, might not be best fit for permanent magnets in manchet)
Steel, low-alloyed {GLO}| market for steel, low-alloyed | Cut-off, S	1,58	g	Screws of air pump
Acrylonitrile-butadiene-styrene copolymer {GLO}| market for acrylonitrile-butadiene-styrene copolymer | Cut-off, S	6,8	g	Plastic case of air pump
Synthetic rubber {GLO}| market for synthetic rubber | Cut-off, S	2	g	Air valves of air pump
Air compressor, screw-type compressor, 4kW {GLO}| market for air compressor, screw-type compressor, 4kW | Cut-off, S	0,000372	p	Proxy process for air compressor. Original compressor process weighs 140kg. Therefore, adjusted for actual weight of compressor 26g (0,026/140). Based on assumed complexity of smaller devices, multiplied chosen dataset by 2.
Steel, low-alloyed {GLO}| market for steel, low-alloyed | Cut-off, S	4,3	g	Air vent connected to compressor tubing, assumed 50% steel = 0.5*8.6g
Electronic component, passive, mobile, earpiece and speaker {GLO}| market for electronic component, passive, mobile, earpiece and speaker | Cut-off, S	4,3	g	Air vent connected to compressor tubing, assumed 50% passive electronic component = 0.5*8.6g
Cable, unspecified {GLO}| market for cable, unspecified | Cut-off, S	20	g	Assumed weight of charging cable
**Electricity/heat**			
Blow moulding {GLO}| market for blow moulding | Cut-off, S	21	g	Inflatable balloon
Metal working, average for steel product manufacturing {GLO}| market for metal working, average for steel product manufacturing | Cut-off, S	30,6	g	Metal oval ring
Injection moulding {GLO}| market for injection moulding | Cut-off, S	23,6	g	Plastic case, upper
Injection moulding {GLO}| market for injection moulding | Cut-off, S	32,2	g	Plastic case, bottom
Injection moulding {GLO}| market for injection moulding | Cut-off, S	9	g	Plastic attachment
Injection moulding {GLO}| market for injection moulding | Cut-off, S	0,4	g	Plastic button

Metal working, average for aluminium product manufacturing {GLO}| market for metal working, average for aluminium product manufacturing | Cut-off, S	0,1	g	Aluminium ring
Extrusion, plastic pipes {GLO}| market for extrusion, plastic pipes | Cut-off, S	1,6	g	Air tubing
Metal working, average for steel product manufacturing {GLO}| market for metal working, average for steel product manufacturing | Cut-off, S	1,58	g	Screws air pump
Injection moulding {GLO}| market for injection moulding | Cut-off, S	6,8	g	Plastic case of air pump
Transport, Road, Bulk/piece, Lorry medium weight - STREAM	132	kgkm	Transport finished product Paris to Leiden (approx 500km) multiplied by total weight product = 263,98g excl packaging
Transport, Road, Bulk/piece, Lorry medium weight - STREAM	530	kgkm	Assumed transport intermediate components Le Havre port to Paris (approx 200). Total weight product = 263,98g excl packaging. Multiplied by factor 10 to account for indirect transport via warehouses and/or intermediary production sites.
Transport, Shipping, Bulk/piece, Deep sea: Bulk carrier 35-60 dwkt - STREAM	50200	kgkm	Assumed transport intermediate components Shanghai to Le Havre (approx 19k km). Total weight product = 263,98g excl packaging. Multiplied by factor 10 to account for indirect transport via warehouses and/or intermediary raw product facilities.
Metal working, average for steel product manufacturing {GLO}| market for metal working, average for steel product manufacturing | Cut-off, S	4,3	g	Air vent steel part
**Emissions to air**			
Carbon dioxide	4,3	kg	Adjustment for upstream transport based on manufacturer data. No further details available, so other impacts had to be disregarded.
**Waste to treatment**			
Waste electric and electronic equipment {GLO}| market for waste electric and electronic equipment | Cut-off, S	263,98	g	

Note to reader

Additional process data is available for each of the subcategories listed in Table B1, yet in Excel file format – and therefore not included in this supplement. Considering the substantial amount of time required to export processes from SimaPro in Excel format and converting them to reader-friendly Word files, the additional files can be obtained from the authors upon reasonable request within a reasonable timeframe. After publication of the study, all processes will be uploaded to the freely accessible HealthcareLCA database (in due time).

Supplement C – Topic list for interviews and focus groups

C1. Interview protocol for healthcare professionals:

Introduction (5min)

a. Welcome and introduction
b. Clarification of subject
c. Agreements and room for questions

a. Welcome and introduction

Thank you for coming and for participating in this follow-up conversation. I suggest that we address each other personally, rather than formally. I will briefly introduce myself and the subject of research, afterwards I would like to hear more about you.

My name is Egid van Bree, I am a physician-researcher in the subject of digital healthcare and sustainability at the LUMC. This research was commissioned by the Dutch National Health Care Institute and is a collaboration between the departments of Cardiology and Public Health and Primary Care.

b. Clarification of subject

In this study and in this conversation, we will discuss your considerations to prescribe or explain telemonitoring in cardiac follow-up – also known as 'the Box'.

c. Agreements and room for questions

I would like to propose and state the following:

- I am curious to hear about your experiences and ideas – there are no wrong answers in my opinion. Please feel free to mention anything that you think of.
- If there are things that you do not understand, please indicate this, so I can do my best to clarify.
- Everything that we discuss today will remain anonymous.
- I will be making notes during our conversation to support the structuring of the conversation. Please do not pay too much attention to how much or how little I am writing and don't feel obliged to wait for me to finish before continuing your sentence.
- The conversation will take approximately 30 minutes and a maximum of 45 minutes.
- I have brought an informed consent form that I would like you to sign.
- To make a transcript of the conversation, I would like to record the interview.

Do you have any questions before we start? Do you have any objections to me recording the interview?

[start recording]

Introduction participant (5min)

Let us start with an introduction. I would like to hear about your role in the department of Cardiology and your reason to participate in this study.

Questions participant (20-30min)

I would like to ask you to base your answers as much as possible on recent conversations which you have had with patients regarding telemonitoring.

1. **What were your considerations to advise patients to use the Box in their follow-up?**
   Probing questions:
   a) Which factors do you weigh?
   b) Which factors or characteristics get most weight?
   c) Which of these factors do you explicitly discuss with patients?

2. **To what extent was the environmental burden or sustainability of the Box included in your advice?**
   Probing questions:
   a) Why is this the case? Or: why not?
   b) Do you find it important to consider sustainability in your choice of medical treatment? Why (not)?

c) How important do you find a more or less sustainable choice, compared to the other factors you mentioned in response to the previous question?

3. **Did you explicitly discuss with patients whether the Box is more or less environmentally sustainable?**
   Based on the participant's answer, focus on their responses to question 4 or 5.

4. **What were the most important reasons that made you less likely to explicitly discuss the environmental sustainability of the Box?**
   Probing questions:
   a) Could you elaborate on these reasons?
   ***b) Knowledge/skills/confidence:** what would you need to be able to do this? Would you expect to discuss it if you did have the knowledge/skills/confidence?*
   ***c) Not important/not my priority:** if not discussed previously: why not important, why not your priority?*
   ***d) Not my role as healthcare professional/not what my colleagues expect of me":** Who do you think should consider the environmental impact of healthcare? Who is expected to do so?*
   ***e) Consultation not suitable/ /too many other things to consider/don't want to change my consultation for this":** why not? What would need to change to make it possible to do so? Would you expect to discuss it if these conditions change?*
   ***f) Fear of negative consequences/negative feelings:** what are these?*
   ***g) Doubts about effectiveness/no benefit of discussing it:** could you elaborate?*
   ***h)** Based on the short online survey, we noticed that colleagues indicated that they had too many other things that they needed to discuss, making it impossible to also consider environmental sustainability. What are your thoughts about this?*

5. **What were the most important reasons that made it likely for you to explicitly discuss the environmental sustainability of the Box?**
   Probing questions:
   a) Could you elaborate on these reasons?
   ***b) Knowledge/skills/confidence:** how did you obtain this knowledge/these skills? Do you expect colleagues to also hold these skills? And patients?*
   ***c) Important/ my priority:** if not discussed previously: why important, why your priority?*
   ***d) My role as healthcare professional/ what my colleagues expect of me":** Why? Who do you think should consider the environmental impact of healthcare?*
   ***e) Consultation suitable/ /possible to consider next to other things/want to change my consultation for this":** why? What makes it possible to do so?*
   ***f) No negative/positive consequences/ feelings:** what are these and why?*
   ***g) Confidence regarding effectiveness/ benefit of discussing it:** could you elaborate?*
   ***h)** Based on the short online survey, we noticed that colleagues indicated that they considered it fitting for their role as healthcare professional to discuss environmental sustainability. What are your thoughts about this?*

6. **Are there other considerations that you would like to share?**

Closing (5min)
Thank you for your answers and for your honesty. These were all the questions that I wanted to discuss with you. In your opinion, are there things that we have not discussed yet or are there additional remarks that you would like to make?

Lastly, I will briefly summarise what we have discussed today. Could you confirm if the most important points are included in my summary?

End of the interview, thanks again.

<u>C2. Focus group/interview protocol for patients:</u>

<u>Introduction</u> (5min)

a. Welcome and introduction
b. Clarification of subject
c. Agreements and room for questions

a. Welcome and introduction

Thank you for coming and for participating in this follow-up conversation. I suggest that we address each other personally, rather than formally. I will briefly introduce myself and the subject of research, afterwards I would like to hear more about you.

My name is Egid van Bree, I am a physician-researcher in the subject of digital healthcare and sustainability at the LUMC. This research was commissioned by the Dutch National Health Care Institute and is a collaboration between the departments of Cardiology and Public Health and Primary Care.

b. Clarification of subject

We have invited you for this study because you have experience with the follow-up after a cardiac event, for which the LUMC also uses remote patient monitoring – also known as 'the Box'. During this interview, I would like to discuss the ideas that you have regarding your usage of remote patient monitoring and the explanation that you have received about this.

c. Agreements and room for questions

I would like to propose and state the following:

- I am curious to hear about your experiences and ideas – there are no wrong answers in my opinion. Please feel free to mention anything that you think of.
- If there are things that you do not understand, please indicate this, so I can do my best to clarify.
- Everything that we discuss today will remain anonymous.
- I will be making notes during our conversation to support the structuring of the conversation. Please do not pay too much attention to how much or how little I am writing and don't feel obliged to wait for me to finish before continuing your sentence.
- The conversation will take approximately 45 minutes and a maximum of 60 minutes.
- I have brought an informed consent form that I would like you to sign.
- To make a transcript of the conversation, I would like to record the interview.

Do you have any questions before we start? Do you have any objections to me recording the interview?

[start recording]

<u>Introduction participants</u> (5-10min)

Let us start with an introduction. I would like to everyone's name, how you are doing today, and why you have chosen to participate in this study.

<u>Questions participants</u> (30-40min)

I would like to ask you to – as much as possible – think back of the situation that you have just been treated for your cardiac condition and are still at the LUMC. The healthcare professional explains to you that you will remain in follow-up (check-ups) for the upcoming year, to make sure that you are recovering well. In the LUMC, this usually takes place via remote patient monitoring – 'the Box'.

1. **Could someone explain what reasons were mentioned to use remote patient monitoring in their cardiac follow-up?**
   Probing questions:
   a) Which reasons to use the Box were discussed with you?
   b) What was important for you in your choice between the Box and care on site? Could you explain why and what you found most important?
   c) Were there reasons why you did or did not want remote monitoring?

For the remainder of this conversation, we will specifically discuss how you perceived the environmental sustainability of the Box – in other words: what is beneficial for nature and the environment – compared to care

on site at the LUMC. We have chosen to only explain this to you now, to make sure that as many different persons as possible would participate in the study – not just people who are specifically interested in environmental sustainability. This way, we can better understand different ideas and opinions.

2. **Was environmental sustainability an argument that was discussed by the physician/nurse and yourself regarding telemonitoring?**
   Probing questions:
   - *a) What exactly was discussed?*
   - *b) How did environmental sustainability influence the decision process?*

3. **What did you think of** [*or*] **what would you have thought of the physician/nurse discussing environmental sustainability as a reason to choose (or not choose) for remote patient monitoring?**
   Probing questions:
   - *a) Could you elaborate? What do you (dis)like about it?*
   - *b) Did this influence* ***how you fee****l about the follow-up? Or your* ***trust*** *in the follow-up? And in what way?*
   - *c) Did this influence your* ***trust in the healthcare professional****? In what way?*
   - *d) Did this influence whether you* ***felt that your health was a priority****? Or your feeling of being listened to?*
   - *e) Is this* ***part of a healthcare professional's role****? Why (not)?*

4. **To what extent did/does environmental sustainability influence your own treatment choice?**
   Probing questions (what were reasons that made it less/more likely to explicitly discuss it):
   - *a)* ***Knowledge/skills****: what would you need to be able to consider it?*
   - *b)* ***Important/my priority****: if not discussed yet: why (not)? Would you accept other disadvantages to make a more environmentally sustainable treatment choice?*
   - *c)* ***Doubts regarding effectivity/no perceived benefit*** *: could you elaborate?*
   - *d)* ***No time during consultation/consultation not suitable to discuss****: why (not)?*
   - *e)* ***Not my role as patient/not expected of me****: who do you think should consider the environmental impact of healthcare? Of whom is it expected to do so?*

5. **Are there other considerations that you would like to share?**

Closing (5min)
Thank you for your answers and for your honesty. These were all the questions that I wanted to discuss with you. In your opinion, are there things that we have not discussed yet or are there additional remarks that you would like to make?

Lastly, I will briefly summarise what we have discussed today. Could you confirm if the most important points are included in my summary?

End of the interview, thanks again.

Supplement D – Life cycle impact assessment and sensitivity/uncertainty analysis for care on site and telemonitoring

Table D1. Life cycle impact assessment for Care on Site

Calculated using "Environmental Footprint 3.1 (adapted) V1.00 / EF 3.1 normalization and weighting set."

Damage category	Unit	Total	Patient commute	Staff commute	Blood tests	Energy use	ECG recording	Cardiac ultrasound	Holter monitoring	Hand desinfection	Computer usage	Examination table paper
Acidification	mol H+ eq	0,04306095	0,029244229	0,003926495	0,00230276	0,00071324	0,00264631	0,00242248	0,00097608	0,00045882	0,00020898	0,00016155
Climate change	kg CO2 eq	11,322759	*7,7535378*	1,0332448	0,52081882	0,6155003	0,50100698	0,62297846	*0,1295369*	*0,0915551*	0,0281825	0,02639761
Ecotoxicity, freshwater	CTUe	120,6195	83,224815	10,657796	3,5042717	0,43821946	7,732385	6,0473729	2,7289277	5,5497712	0,56610353	0,16984236
Particulate matter	disease inc.	5,71E-07	4,20E-07	5,80E-08	2,15E-08	3,82E-09	3,18E-08	1,86E-08	9,10E-09	5,16E-09	1,72E-09	1,64E-09
Eutrophication, marine	kg N eq	0,01287384	0,008611047	0,001143256	0,00059407	0,00020489	0,00063795	0,00134048	0,00016966	7,80E-05	5,03E-05	4,42E-05
Eutrophication, freshwater	kg P eq	0,00182055	0,001183377	0,0001519	0,00012449	1,02E-05	0,00014282	9,89E-05	4,97E-05	2,11E-05	2,77E-05	1,04E-05
Eutrophication, terrestrial	mol N eq	0,11309304	0,078070065	0,01064685	0,0050826	0,00230732	0,00631656	0,00708484	0,00173886	0,00097235	0,00043159	0,000442
Human toxicity, cancer	CTUh	1,03E-08	7,82E-09	1,12E-09	1,75E-10	8,18E-11	4,47E-10	2,00E-10	3,72E-10	3,46E-11	3,67E-11	1,03E-11
Human toxicity, non-cancer	CTUh	1,30E-07	9,61E-08	1,23E-08	2,49E-09	1,85E-09	4,87E-09	3,81E-09	4,45E-09	1,02E-09	2,93E-09	4,08E-10
Ionising radiation	kBq U-235 eq	0,39592039	0,25015428	0,035077736	0,02912858	0,00644348	0,03300173	0,02313227	0,00831525	0,00470014	0,00275158	0,00321535
Land use	Pt	87,466412	62,388146	8,3156493	3,5120272	0,73979401	6,9122715	3,6587081	0,60038374	0,31888184	0,15956064	0,86098891
Ozone depletion	kg CFC11 eq	3,56E-07	1,97E-07	2,54E-08	1,98E-08	2,94E-08	1,63E-08	2,59E-08	4,26E-09	3,66E-08	1,20E-09	6,26E-10
Photochemical ozone formation	kg NMVOC eq	0,04755397	0,035221993	0,005323587	0,00150144	0,00090216	0,00172723	0,00172622	0,00060007	0,00032839	0,00012413	9,87E-05
Resource use, fossils	MJ	158,49704	105,64039	14,142948	8,2010332	10,173674	6,7734198	9,3587981	1,6674064	1,8520912	0,34669517	0,34058221
Resource use, minerals and metals	kg Sb eq	1,30E-04	8,65E-05	1,02E-05	1,10E-05	6,23E-07	3,88E-06	3,09E-06	6,48E-06	1,41E-06	6,25E-06	8,99E-08
Water use	m3 depriv.	3,6113011	1,787783	0,20763769	0,28913757	0,04693279	0,20034385	0,94607773	0,0517355	0,07869716	-0,0010652	0,00402107

Table D2. Life cycle impact assessment for Telemonitoring (using manufacturer LCA data)

Calculated using "Environmental Footprint 3.1 (adapted) V1.00 / EF 3.1 normalization and weighting set."

NB: the watch and scale were NOT included in the SimaPro calculation but added manually based on EF3.0 reporting by the manufacturer (Table B3). Values that were calculated after manual addition are marked in blue. Missing information is marked by 'x'.

Damage category	Unit	Total	Smartwatch	Body scale	BPM	Patient commute	Staff commute	Energy use	Cardiac ultrasound	Digital infrastructure	Blood tests	ECG recording	Holter monitoring	Hand desinfection	Video calling	Examination table paper	Computer usage (clinician)	Laptop usage (patient)
Acidification	mol H+ eq	x	x	x	0,06184553	0,01462212	0,0043927	0,00071324	2,42E-03	0,00138323	0,00136286	0,00132315	0,00097608	0,00022941	1,41E-06	8,08E-05	0,00020898	2,42E-05
Climate change	kg CO2 eq	65,706259	31,9	15,04	11,228424	3,8767689	1,1559256	0,6155003	0,62297846	0,48458921	0,30823971	0,25050349	0,12953685	0,04577753	0,00063172	0,0131988	0,0281825	0,00600152
Ecotoxicity, freshwater	CTUe	x	x	x	210,01988	41,612408	11,923233	0,43821946	6,0473729	1,498704	2,0739567	3,8661925	2,7289277	2,7748856	0,00084887	0,08492118	0,56610353	0,04542183
Particulate matter	disease inc.	2,36E-06	8,57E-07	7,60E-07	3,92E-07	2,10E-07	6,49E-08	3,82E-09	1,86E-08	8,29E-09	1,27E-08	1,59E-08	9,10E-09	2,58E-09	6,85E-12	8,21E-10	1,72E-09	1,99E-10
Eutrophication, marine	kg N eq	x	x	x	1,99E-02	4,31E-03	0,001279	0,00020489	1,34E-03	0,00035173	0,00035159	0,00031898	0,00016966	3,90E-05	3,93E-07	2,21E-05	5,03E-05	5,54E-06
Eutrophication, freshwater	kg P eq	x	x	x	8,19E-03	5,92E-04	1,70E-04	1,02E-05	9,89E-05	3,00E-04	7,37E-05	7,14E-05	4,97E-05	1,06E-05	1,59E-08	5,21E-06	2,77E-05	2,32E-06
Eutrophication, terrestrial	mol N eq	x	x	x	0,13083916	0,03903503	0,01191099	0,00230732	7,08E-03	0,00329833	0,00300807	0,00315828	0,00173886	0,00048618	4,46E-06	0,000221	0,00043159	5,70E-05
Human toxicity, cancer	CTUh	x	x	x	9,09E-09	3,91E-09	1,25E-09	8,18E-11	2,00E-10	1,63E-10	1,03E-10	2,24E-10	3,72E-10	1,73E-11	1,16E-13	5,14E-12	3,67E-11	2,87E-12
Human toxicity, non-cancer	CTUh	x	x	x	3,75E-07	4,81E-08	1,37E-08	1,85E-09	3,81E-09	6,40E-09	1,48E-09	2,44E-09	4,45E-09	5,11E-10	3,48E-12	2,04E-10	2,93E-09	1,57E-10
Ionising radiation	kBq U-235 eq	x	x	x	0,83424801	0,12507714	0,03924264	0,00644348	0,02313227	0,28806835	0,01723936	0,01650087	0,00831525	0,00235007	1,36E-05	0,00160768	0,00275158	0,00035314
Land use	Pt	x	x	x	40,956542	31,194073	9,3029955	0,73979401	3,6587081	1,5720946	2,0785467	3,4561358	0,60038374	0,15944092	0,001532	0,43049445	0,15956064	0,01818483

Ozone depletion	kg CFC11 eq	x	x	x	1,22E-06	9,83E-08	2,85E-08	2,94E-08	2,59E-08	1,19E-08	1,17E-08	8,14E-09	4,26E-09	1,83E-08	2,05E-11	3,13E-10	1,20E-09	2,33E-10
Photochemical ozone formation	kg NMVOC eq	x	x	x	0,03834835	0,017611	0,00595568	0,00090216	1,73E-03	0,00104467	0,00088861	0,00086362	0,00060007	0,0001642	1,46E-06	4,94E-05	0,00012413	1,74E-05
Resource use, fossils	MJ	930,6	511,0	214,1	93,62815	52,820197	15,82219	10,173674	9,3587981	12,316794	4,8536727	3,3867099	1,6674064	0,9260456	0,01052607	0,1702911	0,34669517	0,08940412
Resource use, minerals and metals	kg Sb eq	0,00585	0,00130	0,00241	2,05E-03	4,32E-05	1,15E-05	6,23E-07	3,09E-06	4,41E-06	6,50E-06	1,94E-06	6,48E-06	7,06E-07	1,17E-09	4,49E-08	6,25E-06	4,64E-07
Water use	m3 depriv.	16,33	4,50	7,677419355	1,393311	0,8938915	0,23229125	0,04693279	0,94607773	0,27418146	0,17112223	0,10017192	0,0517355	0,03934858	9,84E-05	0,00201053	-0,0010652	0,00018347

Table D3. Manufacturer EF 3.0 reporting for devices (reference table for Table B2)

"Use phase" correction calculated based on manufacturer LCA results; calculated group needs to be subtracted from total impact							
Damage category	**Smartwatch**	*Total*	*Use phase (subtraction)*	**Body scale**	*Total*	*Use phase (subtraction)*	
Climate change		32,2	0,3		16,4	1,36	*NB: body scale has a 5 year use phase in manufacturer LCA data*
Particulate matter		8,68E-07	1,08446E-08		7,79E-07	1,87E-08	*NB: smartwatch has a 2 year use phase in manufacturer LCA data*
Resource use, fossils		516,1	5,161290323		223,0	8,921212121	
Resource use, minerals and metals		0,00130	0		0,00246	4,92823E-05	
Water use		4,51	0,011282051		8,06451613	0,387096774	

Table D4. Life cycle impact assessment for Telemonitoring (using own disassembly data)

Calculated using "Environmental Footprint 3.1 (adapted) V1.00 / EF 3.1 normalization and weighting set."

NB: The watch and scale were included in the SimaPro calculation based on own disassembly data; the watch (approx 50%) and scale (approx 75%) had lower impacts than manufacturer data.

Damage category	Unit	Total	Smartwatch	Body scale	BPM	Patient commute	Staff commute	Energy use	Cardiac ultrasound	Digital infrastructure	Blood tests	ECG recording	Holter monitoring	Examination table paper	Hand desinfection	Computer usage (clinician)	Video calling	Laptop usage (patient)
Acidification	mol H+ eq	0,2929217	0,074194653	0,12914093	0,06184553	0,01462212	0,0043927	0,00071324	0,00242248	0,00138323	0,00136286	0,00132315	0,00097608	8,08E-05	0,00022941	0,00020898	1,41E-06	2,42E-05
Climate change	kg CO2 eq	48,317479	14,672679	14,878541	11,228424	3,8767689	1,1559256	0,6155003	0,62297846	0,48458921	0,30823971	0,25050349	0,12953685	0,0131988	0,04577753	0,0281825	0,00063172	0,00600152
Ecotoxicity, freshwater	CTUe	655,27207	88,546596	283,0444	210,01988	41,612408	11,923233	0,43821946	6,0473729	1,498704	2,0739567	3,8661925	2,7289277	0,08492118	2,7748856	0,56610353	0,00084887	0,04542183
Particulate matter	disease inc.	1,69E-06	3,19E-07	6,33E-07	3,92E-07	2,10E-07	6,49E-08	3,82E-09	1,86E-08	8,29E-09	1,27E-08	1,59E-08	9,10E-09	8,21E-10	2,58E-09	1,72E-09	6,85E-12	1,99E-10
Eutrophication, marine	kg N eq	0,07987002	0,013085903	0,038478	1,99E-02	0,00430552	0,001279	0,00020489	0,00134048	0,00035173	0,00035159	0,00031898	0,00016966	2,21E-05	3,90E-05	5,03E-05	3,93E-07	5,54E-06
Eutrophication, freshwater	kg P eq	0,02856621	0,010306953	0,008661145	8,19E-03	0,00059169	0,00016994	1,02E-05	9,89E-05	0,00030031	7,37E-05	7,14E-05	4,97E-05	5,21E-06	1,06E-05	2,77E-05	1,59E-08	2,32E-06
Eutrophication, terrestrial	mol N eq	0,73983753	0,12653813	0,40971831	0,13083916	0,03903503	0,01191099	0,00230732	0,00708484	0,00329833	0,00300807	0,00315828	0,00173886	0,000221	0,00048618	0,00043159	4,46E-06	5,70E-05
Human toxicity, cancer	CTUh	3,38E-08	5,84E-09	1,25E-08	9,09E-09	3,91E-09	1,25E-09	8,18E-11	2,00E-10	1,63E-10	1,03E-10	2,24E-10	3,72E-10	5,14E-12	1,73E-11	3,67E-11	1,16E-13	2,87E-12
Human toxicity, non-cancer	CTUh	1,16E-06	2,13E-07	4,82E-07	3,75E-07	4,81E-08	1,37E-08	1,85E-09	3,81E-09	6,40E-09	1,48E-09	2,44E-09	4,45E-09	2,04E-10	5,11E-10	2,93E-09	3,48E-12	1,57E-10
Ionising radiation	kBq U-235 eq	6,5359324	3,7397345	1,4308545	0,83424801	0,12507714	0,03924264	0,00644348	0,02313227	0,28806835	0,01723936	0,01650087	0,00831525	0,00160768	0,00235007	0,00275158	1,36E-05	0,00035314
Land use	Pt	171,48941	19,379026	57,781895	40,956542	31,194073	9,3029955	0,73979401	3,6587081	1,5720946	2,0785467	3,4561358	0,60038374	0,43049445	0,15944092	0,15956064	0,001532	0,01818483

Ozone depletion	kg CFC11 eq	2,28E-06	2,43E-07	5,82E-07	1,22E-06	9,83E-08	2,85E-08	2,94E-08	2,59E-08	1,19E-08	1,17E-08	8,14E-09	4,26E-09	3,13E-10	1,83E-08	1,20E-09	2,05E-11	2,33E-10
Photochemical ozone formation	kg NMVOC eq	0,23034615	0,046574313	0,11547497	0,03834835	0,017611	0,00595568	0,00090216	0,00172622	0,00104467	0,00088861	0,00086362	0,00060007	4,94E-05	0,0001642	0,00012413	1,46E-06	1,74E-05
Resource use, fossils	MJ	649,39202	233,63138	210,19009	93,62815	52,820197	15,82219	10,173674	9,3587981	12,316794	4,8536727	3,3867099	1,6674064	0,1702911	0,9260456	0,34669517	0,01052607	0,08940412
Resource use, minerals and metals	kg Sb eq	0,00481002	0,000884429	0,001788102	2,05E-03	4,32E-05	1,15E-05	6,23E-07	3,09E-06	4,41E-06	6,50E-06	1,94E-06	6,48E-06	4,49E-08	7,06E-07	6,25E-06	1,17E-09	4,64E-07
Water use	m3 depriv.	10,159856	3,340196	2,669369	1,393311	0,8938915	0,23229125	0,04693279	0,94607773	0,27418146	0,17112223	0,10017192	0,0517355	0,00201053	0,03934858	-0,0010652	9,84E-05	0,00018347

Table D5. Environmental impact comparison of Care on Site and Telemonitoring
Comparison based on impact assessment using own disassembly data (Table B4), except for those outcomes where manufacturer information was available, marked in blue (Table B2).

Damage category	Unit	Total CoS	Total RPM	Difference (RPM = 'n' times larger)
Acidification	mol H+ eq	0,04241268	0,2909911	6,9
Climate change	kg CO2 eq	11,322759	65,706259	5,8
Ecotoxicity, freshwater	CTUe	120,17999	651,81909	5,4
Particulate matter	disease inc.	5,69E-07	2,36E-06	4,1
Eutrophication, marine	kg N eq	0,01266314	0,079278349	6,3
Eutrophication, freshwater	kg P eq	0,00181098	0,028512331	15,7
Eutrophication, terrestrial	mol N eq	0,11071533	0,73377803	6,6
Human toxicity, cancer	CTUh	1,03E-08	3,34E-08	3,3
Human toxicity, non-cancer	CTUh	1,30E-07	1,15E-06	8,9
Ionising radiation	kBq U-235 eq	0,38795902	6,5159289	16,8
Land use	Pt	87,357791	169,09239	1,9
Ozone depletion	kg CFC11 eq	3,46E-07	2,26E-06	6,5
Photochemical ozone formation	kg NMVOC eq	0,04675876	0,22784203	4,9
Resource use, fossils	MJ	152,78932	930,6473828	6,1
Resource use, minerals and metals	kg Sb eq	0,00013041	0,005853451	44,9
Water use	m3 depriv.	3,5716487	16,32924892	4,6

Table D6. Sensitivity analysis of allocation choices for telemonitoring devices c.q. tailored use of devices
NB: the different scenarios for allocation also function as redistribution of the eHealth devices/re-use for multiple patients, assuming the device cleaning impact to be negligible.

The environmental impact outcomes of the RPM scenario are based on manufacturer data for smartwatch and body scale and own data for BPM and other processes. Since no further manufacturer data was available, only the outcomes climate change, particulates, fossil use, minerals and metals use, and water deprivation were included. Orange numbers are not available since not further 'simple watch' data were available.

			1x redistributed	2x redistirbuted	3x redistributed				
Damage category	Unit	100% allocation (reference scenario)	50% allocation	33% allocation	25% allocation	Smartwatch only (100% allocation)	Simple watch + BPM (100% allocation)	Simple watch + BPM (50% allocation)	BPM only (100% allocation)
Climate change	kg CO2 eq	65,7	36,6	26,9	22,1	39,4	25,8	20,2	18,8
Particulate matter	disease inc.	2,4E-06	1,4E-06	1,0E-06	8,5E-07	1,2E-06	n/a	n/a	7,4E-07
Resource use, fossils	MJ	930,6	521,3	384,8	316,6	622,9	n/a	n/a	205,6
Resource use, minerals and metals	kg Sb eq	5,9E-03	3,0E-03	2,0E-03	1,5E-03	1,4E-03	n/a	n/a	2,1E-03
Water use	m3 depriv.	16,3	9,5	7,3	6,2	7,3	n/a	n/a	4,2

Tables D7a/b. Sensitivity analysis of different one-way commute distances and means of transportation
NB: the different scenarios for travel distance and means of travel also function as an exploration of: 1) the 'Break Even Point' for CoS = RPM; 2) use of RPM in rural vs city settings.

The environmental impact outcomes of the RPM scenario are based on manufacturer data for smartwatch and body scale and own data for BPM and other processes. Since no further manufacturer data was available, only the outcomes climate change, particulates, fossil use, minerals and metals use, and water deprivation were included.

Table D7a. Telemonitoring

Damage category	Unit	7 km one way, 50% car	7 km one way, 100% car	14 km one way, 100% car	28 km one way, 100% car	56 km one way, 100% car	65 km one way, 100% car	84 km one way, 100% car
Climate change	kg CO2 eq	65,7	68,2	74,6	87,3	112,8	120,9	138,2
Particulate matter	disease inc.	2,4E-06	2,5E-06	2,9E-06	3,6E-06	5,0E-06	5,4E-06	6,4E-06
Resource use, fossils	MJ	930,6	963,8	1049,8	1221,7	1565,6	1676,1	1909,4
Resource use, minerals and metals	kg Sb eq	5,9E-03	5,9E-03	6,0E-03	6,1E-03	6,5E-03	6,6E-03	6,8E-03
Water use	m3 depriv.	16,3	17,1	18,8	22,1	28,8	30,9	35,5

Table D7b. Care on Site

Damage category	Unit	7 km one way, 50% car	7 km one way, 100% car	14 km one way, 100% car	28 km one way, 100% car	56 km one way, 100% car	65 km one way, 100% car	84 km one way, 100% car
Climate change	kg CO2 eq	11,3	16,3	29,0	54,5	105,4	121,8	156,3
Particulate matter	disease inc.	5,7E-07	8,6E-07	1,6E-06	3,0E-06	5,8E-06	6,7E-06	8,6E-06
Resource use, fossils	MJ	158,5	224,8	396,7	740,6	1428,3	1649,4	2116,1
Resource use, minerals and metals	kg Sb eq	1,3E-04	2,0E-04	3,6E-04	6,8E-04	1,3E-03	1,5E-03	2,0E-03
Water use	m3 depriv.	3,6	5,2	8,5	15,2	28,5	32,8	41,9

Table D8. Sensitivity analyses to test the effect on outcomes of data choices and assumptions

Category	*Explanation*	*Type*	*Source*	*Effect*		
**Commute / individual travel**	Dutch average car transportation mix for employees and patients (Ecoinvent) rather than the Netherlands-specific STREAM transportation data	Alternative data choice	main contributing process to Care on Site scenario, largest contributor to RPM scenario after eHealth devices	Alteration influences both patient commute (main effect) and staff commute (minor effect, due to alloation). Notable effect on climate change, particulate matter, and fossil resource use. Increase of e.g. climate change large for CoS (+43%; +4.7kg CO2eq) and minor for RPM (+4%; +2.6kg CO2 eq). Considering the large impact of eHealth devices, does not alter the conclusion that RPM>CoS. Does influence the 'break even point' for patient commute distance. Now at approx 45-50km one way, rather than 65km one way.		
**Energy source**	Dutch average electricity mix (Ecoinvent) rather than the Netherlands-specific energy mix based on CE Delft data (2021): the NL mix contains more fossil fuel based electricity generation	Alternative data choice	minor contributing process in both CoS and RPM, known effect of data choice	Alteration mainly influences energy use for both scenario's, but also affects digital infrastructure (RPM) due to energy consumption for server storage, data transfer, and video calling. Minor effect on climate change, particulate matter, and fossil resource use. Increase of e.g. climate change for CoS (+5%; +0.5 kg CO2eq) and RPM (+1%; +0.7 kg CO2eq). No substantial effect on comparison or break even point.		
**Data transfer 'PUE'**	power usage effectiveness (PUE) of data transfer and data storage in kWh/GB is used from Swiss Federal Office for Energy (2020 data adjusted for 2023) or Sillcox et al (2023) rather than the Jackson et al (2023) figures	Alternative data choice	minor contributing proces, verify if impact in RPM scenario would be substantially larger	Alteration only influences the RPM scenario. PUEs of respectively 0.0075 kWh/GB (Jackson), 0.113 kWh/GB (SFOE), and 0.64 kWh/GB (Sillcox). Effects on outcomes <1% and therefore no effect on comparison etc.		

Note to reader

NB: variance of staff commute and energy use in both CoS and RPM were already included in the uncertainty analysis (Monte Carlo) by predefining possible ranges for the number of patients seen in a day, average surface area of the outpatient clinic rooms, and variations in potential data size (storage) required for usage of RPM. Therefore no separate sensitivity analysis has been performed.

Figure D1. Uncertainty analysis of the Care on Site and Telemonitoring comparison
Executed in SimaPro v9.5.0.1 using Monte Carlo simulations for 1,000 runs; 95% confidence interval.

NB: the watch and scale were included in the SimaPro calculation based on own disassembly data; the watch (approx 50%) and scale (approx 75%) had lower impacts than manufacturer data.

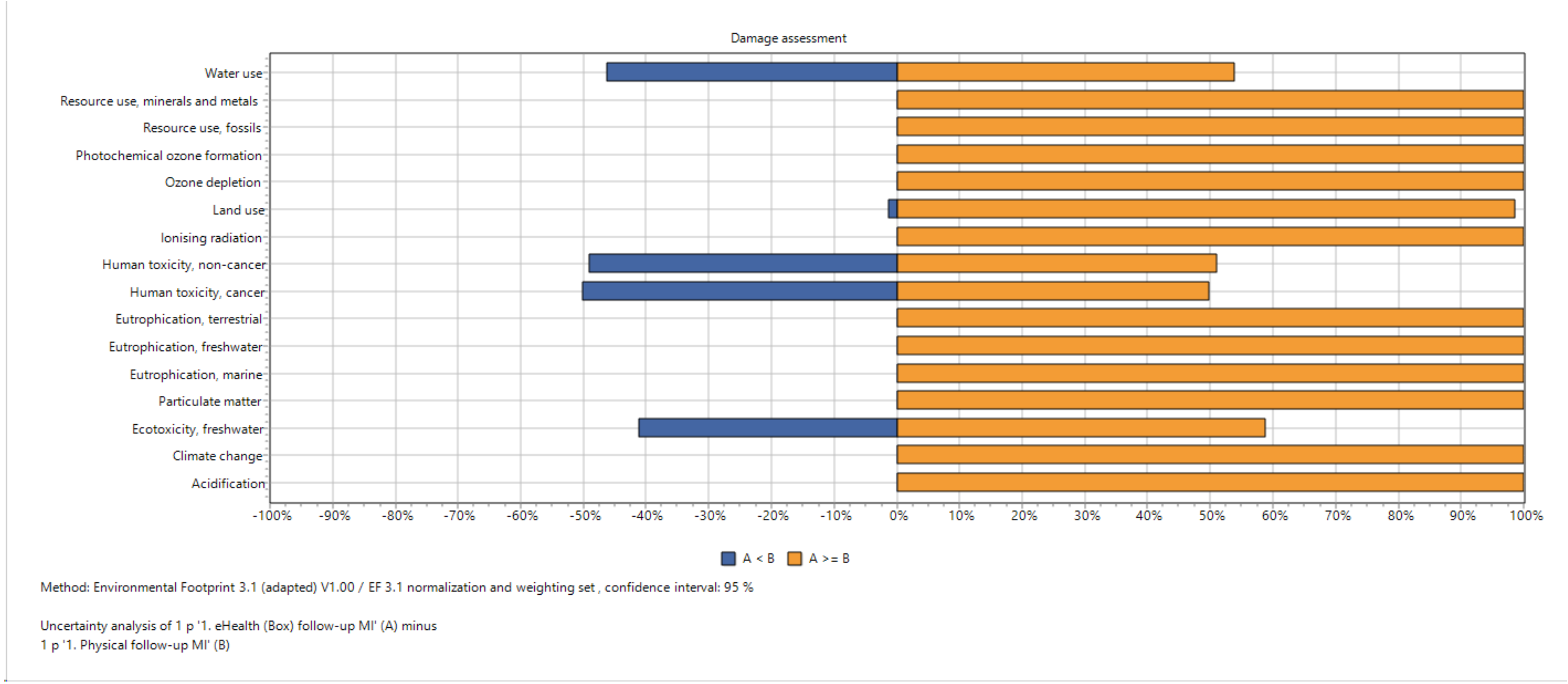

NB: in the comparison above, for both scenarios the patient commute is varied using a lognormal distribution for the mean distance of 14 km both ways and the means of transport is fixed on 50% car, 25% (e)bike, and 25% public transport.
NB: for both scenarios, the staff commute is altered based on the number of patients seen per working day (20-40) and a lognormal distribution of the mean 35.6 km distance (both ways) of staff commute; the means of travel are fixed based on the internal audit.

NB: for both scenarios, the energy use is altered based on the number of patients (15-30), the outpatient room surface area (12-20 m2), and the computed ranges for LUMC energy usage values.
NB: for the RPM scenario, the device allocation is fixed at a functional allocation of 100%, also meaning that NO reuse takes place.

Note: no uncertainty analysis for the comparison has been made for scenarios where allocation choices for the devices or patient travel distance are substantially larger, considering that the sensitivity analysis already explores the influence of different allocation choices (c.q. reuse) and longer commute distances.

In addition: no uncertainty analysis has been made for the actual manufacturer LCA data, since no (reliable) uncertainty ranges were available or could be computed. Moreover, the comparison above already showed a clear difference in environmental impact between RPM and CoS based on the RPM scenario using own data (which has a lower environmental impact than the actual products).

Table D9. Uncertainty analysis of the Care on Site and Telemonitoring comparison
Executed in SimaPro v9.5.0.1 using Monte Carlo simulations for 1,000 runs; 95% confidence interval.

Damage category	A >= B	Mean	Median	SD	CV	2,50%	97,50%	SEM
Acidification	100	0,25772329	0,25593516	0,02545741	9,8778067	0,21279086	0,31234097	0,00080503
Climate change	100	39,596746	39,441082	3,7109997	9,3719815	33,148905	46,981304	0,11735211
Ecotoxicity, freshwater	58,8	793,08368	870,28741	4295,2421	541,5875	-7261,8207	9039,611	135,82748
Eutrophication, freshwater	100	0,02830159	0,02627651	0,01032509	36,482377	0,01561025	0,05275511	0,00032651
Eutrophication, marine	100	0,06902401	0,06828768	0,00816765	11,833061	0,05514341	0,08571754	0,00025828
Eutrophication, terrestrial	100	0,6448975	0,64039833	0,07779063	12,062479	0,51862941	0,80534915	0,00245996
Human toxicity, cancer	49,8	-1,78E-07	-4,02E-08	6,14E-06	-3456,275	-1,29E-05	1,21E-05	1,94E-07
Human toxicity, non-cancer	51	-1,09E-05	1,51E-05	0,00078655	-7209,0834	-0,0016589	0,00151669	2,49E-05
Ionising radiation	100	8,0044787	5,431372	9,2546525	115,61843	2,6548301	29,049496	0,29265781
Land use	98,6	91,145345	90,534569	39,657217	43,509865	15,78147	171,48463	1,2540713
Ozone depletion	100	2,00E-06	1,94E-06	4,21E-07	20,992585	1,37E-06	3,06E-06	1,33E-08
Particulate matter	100	1,19E-06	1,07E-06	6,88E-07	57,751938	6,96E-07	2,38E-06	2,18E-08
Photochemical ozone formation	100	0,18921059	0,18728902	0,0216274	11,430332	0,15227234	0,23528567	0,00068392
Resource use, fossils	100	559,96345	557,19514	82,050813	14,652887	410,55896	738,30822	2,5946745
Resource use, minerals and metals	100	0,0047117	0,00448038	0,00135469	28,751536	0,00261019	0,00784733	4,28E-05
Water use	53,8	25,79897	73,961768	881,38923	3416,3738	-1907,9864	1598,3608	2,79E+01

References

1. Treskes RW, van Winden LAM, van Keulen N, et al. Effect of Smartphone-Enabled Health Monitoring Devices vs Regular Follow-up on Blood Pressure Control Among Patients After Myocardial Infarction: A Randomized Clinical Trial. *JAMA Netw Open*. 2020;**3**(4):e202165.
2. Berg, van den R, Seters, van D. STREAM personenvervoer 2023. Delft: *CE Delft*; 2024.
3. Kleijn A, Hilster D, Otten M, Scholten P. STREAM Freight Transport 2020. Delft: *CE Delft*; 2021.
4. McAlister S, McGain F, Petersen M, et al. The carbon footprint of hospital diagnostic imaging in Australia. *Lancet Reg Health West Pac*. 2022;**24**:100459
5. McAlister S, Grant T, McGain F. An LCA of hospital pathology testing. *Int J Life Cycle Ass*. 2021;**26**:1753–63
6. Spoyalo K, Lalande A, Rizan C, et al. Patient, hospital and environmental costs of unnecessary bloodwork: capturing the triple bottom line of inappropriate care in general surgery patients. *BMJ Open Qual*. 2023;**12**:e002316
7. Olivetti E, Gregory J, Kirchain R. Life cycle impacts of alkaline batteries with a focus on end-of-life. Cambridge (MA): *Massachusetts Institute of Technology*; 2011.
8. Bruinsma M, Nauta M. Ketenemissies elektriciteit: Acutalisatie elektriciteitsmix 2021. Delft: *CE Delft*; 2023.
9. Thiel CL, Mehta N, Sejo CS, et al. Telemedicine and the environment: life cycle environmental emissions from in-person and virtual clinic visits. *NPJ Digit Med*. 2023;**6**(1):87.
10. Jackson T, Hodgkinson IR. Is there a role for knowledge management in saving the planet from too much data? *Knowl Manag Res Pract.* 2023;**21**(3):427–35
11. Coroama V. Investigating the Inconsistencies among Energy and Energy Intensity Estimates of the Internet. Bern: *Swiss Federal Office of Energy SFOE*; 2021.
12. Koho Enterprise Solid-State Drive Product Life Cycle Assessment Summary. USA: *Seagate Technology LLC*; 2016.
13. Tannu S, Nair PJ. The Dirty Secret of SSDs: Embodied Carbon. ACM SIGENERGY Energy Inf Rev. 2023;**3**(3):4–9
